# Multivalent interactions drive nucleosome binding and efficient chromatin deacetylation by SIRT6

**DOI:** 10.1038/s41467-020-19018-y

**Published:** 2020-10-16

**Authors:** Wallace H. Liu, Jie Zheng, Jessica L. Feldman, Mark A. Klein, Vyacheslav I. Kuznetsov, Craig L. Peterson, Patrick R. Griffin, John M. Denu

**Affiliations:** 1 Department of Biomolecular Chemistry, University of Wisconsin-Madison, Madison, WI 53706 USA; 2 Wisconsin Institute for Discovery, University of Wisconsin-Madison, Madison, WI 53715 USA; 3 Department of Molecular Medicine, Scripps Research Florida, Jupiter, FL 33458 USA; 4 Program in Molecular Medicine, University of Massachusetts Medical School, Worcester, MA 01605 USA

**Keywords:** Chromatin, Nucleosomes, Acetylation

## Abstract

The protein deacetylase SIRT6 maintains cellular homeostasis through multiple pathways that include the deacetylation of histone H3 and repression of transcription. Prior work suggests that SIRT6 is associated with chromatin and can substantially reduce global levels of H3 acetylation, but how SIRT6 is able to accomplish this feat is unknown. Here, we describe an exquisitely tight interaction between SIRT6 and nucleosome core particles, in which a 2:1 enzyme:nucleosome complex assembles via asymmetric binding with distinct affinities. While both SIRT6 molecules associate with the acidic patch on the nucleosome, we find that the intrinsically disordered SIRT6 C-terminus promotes binding at the higher affinity site through recognition of nucleosomal DNA. Together, multivalent interactions couple productive binding to efficient deacetylation of histones on endogenous chromatin. Unique among histone deacetylases, SIRT6 possesses the intrinsic capacity to tightly interact with nucleosomes for efficient activity.

## Abstract (web summary)

SIRT6 plays essential roles in metabolism, tumor suppression, and DNA repair through the deacetylation of histone substrates. Here the authors use biophysical methods to investigate the molecular basis for SIRT6 interaction with the nucleosome core particle.

## Introduction

Recent animal studies reveal that the NAD^+^-dependent deacylase SIRT6 promotes longevity, attributed to homeostatic roles in multiple pathways^1,2^. SIRT6 knockout mice die several weeks after experiencing hypoglycemia, osteopenia, and lymphopenia, while SIRT6-deficient monkeys only live several hours after birth, experiencing similar defects^3,4^. Overexpression of SIRT6 or SIRT6 isoforms with higher activity lead to longevity phenotypes in various rodent models^5,6^. Functionally, SIRT6 removes specific fatty acyl groups from substrates, most notably acetyl modifications from histone H3^7–9^. Histone deacetylation drives repression of a host of glycolytic genes and oncogenic c-myc and NF-ΚB^10,11^. In addition to deacylation activity, SIRT6 promotes DNA repair pathways through ADP-ribosylation of PARP-1, as well as recruitment of the chromatin remodellers SNF2H and CHD4^12–14^. Thus, functional SIRT6 attends to the upkeep of myriad pathways that maintain metabolism and tumor suppression.

The collective biological insight into SIRT6 function reveals that enzyme activity is linked to cellular homeostasis. Multiple studies show, however, that SIRT6 deacetylates lysine 9 on histone H3 (H3K9ac) peptides with a turnover rate on the minutes scale, ~3 orders of magnitude slower than other sirtuins^8,15^. This poor in vitro activity does not reconcile with observations in biology, suggesting that peptide-independent mechanisms promote more robust SIRT6 activity. Importantly, histone peptides only represent a potentially small portion of the interaction surface present on nucleosomes, the fundamental repeating unit of chromatin structure. Nucleosome particles consist of a tetrameric unit of histones (H3/H4)_2_ flanked by two H2A/H2B dimers, forming an octameric core that is enveloped by ~147 base pairs of superhelical DNA^16^. Proteins bind nucleosomes through various docking sites, including acidic patches presented by each of the H2A/H2B dimers and DNA regions of various curvatures^17^. Although many histone deacetylases are members of multi-subunit complexes that possess separate nucleosome-binding proteins^18^, SIRT6 is not known to be part of such complexes. Instead, SIRT6 has been observed to directly bind heterogenous nucleosomes in vitro, and is found almost exclusively in the chromatin sub-fraction of the nucleus^3,19^. Importantly, SIRT6 alone incubated with reconstituted nucleosomes leads to faster H3K9ac deacetylation than parallel experiments with histones alone^20^, suggesting that SIRT6 might intrinsically bind nucleosomes with high affinity. Thus, we reason that tight, specific SIRT6-nucleosome interactions promote improved enzyme efficacy that endows SIRT6 with the capacity to globally reduce H3 acetylation.

Surprisingly, a rigorous biochemical and thermodynamic understanding of the mechanisms driving SIRT6 interactions with nucleosomes is lacking. To uncover the underlying principles that lead to assembly of a SIRT6:nucleosome complex, and how this binding is coupled to efficient histone deacetylation, we investigate the complex with biophysical and biochemical tools. We show that SIRT6 associates with nucleosomes through two asymmetric binding sites using multiple contact points, including an intrinsically disordered C-terminus that associates with nucleosomal DNA. Together, these multivalent interactions promote high affinity engagement with substrate, leading to productive deacetylation in cells. Unlike most histone deacetylases and acetyltransferases that require multi-subunit complexes, our studies reveal that SIRT6 harbors the intrinsic capacity to bind tightly and efficiently catalyze the removal of acetyl-groups from nucleosomes without the need to form elaborate protein complexes.

## Results

### Two SIRT6 molecules engage a nucleosome core particle

**Fig. 1:**
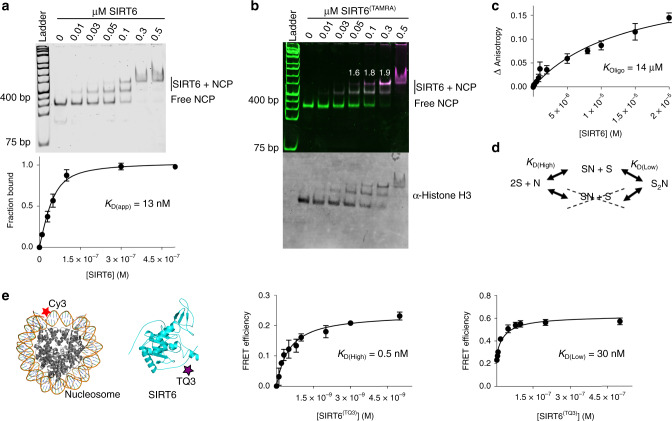
SIRT6 and NCPs assemble with low nanomolar affinity via a two-site binding mechanism. **a** EMSA of NCPs bound to varying SIRT6 concentrations, then detected by SYBR Safe fluorescence. Data are presented as the mean ± s.d. from six independent experiments. The apparent *K*_D_ was calculated using a ligand-depleted equation. **b** SIRT6:NCP stoichiometry was determined by detecting fluorescence from both SYBR Safe-stained DNA and TAMRA-labeled SIRT6. The white numbers indicate the relative TAMRA signal in the higher shifted band compared to the lower bound band, both normalized to DNA content. The gel was subsequently blotted for histone H3. The image is representative of three independent experiments. **c** To measure SIRT6 self-oligomerization, the fluorescence anisotropy of pyrene-labeled SIRT6 was monitored with titration of unlabeled SIRT6. Data are presented as mean ± s.d. from three independent experiments. **d** The data indicates a two-site binding mechanism, in which two SIRT6 molecules bind at separate sites on a nucleosome. **e** A FRET assay was employed to measure binding between 1 nM of Cy3-labeled nucleosomes and SIRT6 labeled with TQ3, which accepts energy transfer from Cy3. The titration reveals a two-site binding curve (Supplementary Fig. 2f). The intermediate binding constant *K*_D(High)_ was fitted to a ligand-depleted equation from titration points up to 5 nM SIRT6^(TQ3)^, while *K*_D(Low)_ was fitted to a specific-binding equation from data points ranging from 5 nM to 500 nM. Data are presented as mean ± s.d. from four independent experiments. Source data are provided as a source data file.

SIRT6 activity appears more robust when endogenously-sourced nucleosomes, rather than histone peptides, are used as substrates^20^. This suggests that direct binding between SIRT6 and nucleosomes is thermodynamically more favorable than interactions with histones and histone peptides, explaining the apparent faster rates of deacetylation. To quantitatively assess the strength of nucleosome binding, we analyzed the complexes formed in electrophoretic mobility shift assays (EMSAs) when purified SIRT6 was added to recombinant 601-positioned nucleosome core particles (NCPs; Fig. 1a and Supplementary Fig. 1a)^21^. Notably, two distinct, bound complexes were evident as SIRT6 was titrated into a fixed level of NCPs: the lower bound complex (faster migrating) appeared and saturated at much lower SIRT6 concentrations, while the slower migrating species appeared at higher concentrations of SIRT6 (Fig. 1a). To determine apparent binding affinity, we used both shifted complexes together as the bound fraction, and the free NCP band as the unbound fraction. The composite affinity is exceptionally tight (*K*_D(app)_ = 13 nM; Fig. 1a and Table 1), enabling the complex to remain fully bound even in the presence of high ionic strength (Supplementary Fig. 2a). Importantly, the low nanomolar affinity observed for NCPs is unprecedented for deacetylases, indicative of novel binding modes exclusive to SIRT6.

**Table 1:** *K*_D_ and IC_50_ values determined in this study.

Protein or complex	Binding partner	EMSA *K*_D_ or IC_50_ (M)	Fluorescence *K*_D_ (M)
NCP	SIRT6	1.3 ± 0.2 × 10^−8^	–
NCP^(Cy3)^	SIRT6^(TQ3)^	–	*K*_D(High)_ 4.6 ± 0.3 × 10^−10^
			*K*_D(Low)_ 3.0 ± 0.9 × 10^−8^
NCP^(AP)^	SIRT6	1.4 ± 0.2 × 10^−7^	–
NCP	SIRT6 + SIRT6^(1–292)^	K_D(Low)_ 4.0 ± 1.4 × 10^−8^	–
NCP	SIRT6^(AAA)^	9.7 ± 0.6 × 10^−8^	–
NCP	SIRT6^(1–292)^	2.5 ± 0.1 ×10^−7^	–
NCP	SIRT6^(1–301)^	1.4 ± 0.1 × 10^−7^	–
NCP	SIRT6^(270–355)^	4.0 ± 0.1 × 10^−7^	–
DNA	SIRT6^(270–355)^	4.5 ± 0.1 × 10^−7^	–
NCP:SIRT6	LANA	9.5 ± 4.5 × 10^−6^	–
NCP:SIRT6^(1–292)^	LANA	6.1 ± 2.1 × 10^−6^	–
NCP:SIRT6^(270–355)^	LANA	n.i.	–
SIRT6^(Py)^	SIRT6	–	1.4 ± 0.6 × 10^−5^

*n.i.* no inhibition.

*K*_D_ and IC_50_ values for each interaction studied (at least three independent experiments) were calculated as described in the Methods section. Curve-fitting errors are reported for each value.

The observation of two separate complexes formed between SIRT6 and NCPs suggested possible assembly into a final 2:1 SIRT6:nucleosome configuration, or that two 1:1 complexes with distinct conformations can form. To differentiate between these possibilities, we labeled SIRT6 with tetramethylrhodamine (TAMRA) at cysteine 320 (SIRT6^(TAMRA)^) and performed nucleosome-binding experiments (Fig. 1b). After scanning the TAMRA signal, we also imaged SYBR Safe-stained DNA and immunoblotted histone H3. The merged fluorescent image showed that the slowest migrating complex indeed has twice the TAMRA signal of the faster complex, providing evidence that two SIRT6 molecules occupy a single, intact nucleosome (Fig. 1b and Supplementary Fig. 2b).

The 2:1 complex might be formed through nucleosome bridging, or the individual SIRT6 molecules might dimerize prior to nucleosome encounter. To determine if dimerization is a possible mechanism, we monitored the oligomerization of SIRT6 through fluorescence anisotropy. We prepared SIRT6 labeled with pyrene at cysteine 18 (SIRT6^(Py)^), then measured pyrene anisotropy with titration of unlabeled SIRT6 (Fig. 1c). The resulting isotherm showed that SIRT6 indeed increased the anisotropy signal, but with an oligomerization constant 1000x weaker than the affinity of SIRT6:NCP (Fig. 1a and Table 1). Thus, SIRT6 is unlikely to oligomerize before binding nucleosomes, consistent with the EMSA results, as a dimerization mechanism would not produce a 1:1 SIRT6:NCP complex in native gels (Fig. 1a). Together, assembly of a high affinity 2:1 SIRT6:NCP complex requires two binding sites bridged by the nucleosome.

The appearance of a 1:1 complex in the SIRT6 titration of NCPs suggested that the two binding events likely involve a high affinity site and a low affinity one (Fig. 1d). To confirm this equilibrium model and measure the individual binding constants in solution, we designed a more sensitive FRET assay. We reconstituted nucleosomes with Cy3 positioned at the first nucleotide of the DNA entry/exit site, and prepared SIRT6 labeled with Tide Quencher 3 (TQ3) at cysteine 18 (SIRT6^(TQ3)^), providing the enzyme with a dark acceptor fluorophore for Cy3 emission (Fig. 1e and Supplementary Fig. 2c–f). The Förster radius of the Cy3-TQ3 FRET pair was calculated to be 38.1 Å (Methods section). Upon titrating SIRT6^(TQ3)^ to Cy3-labeled NCPs, we observed maximum FRET efficiency at 57%, which corresponded to an average distance of ~36 Å between the labeled cysteines and the Cy3-labeled nucleotide. The binding isotherm revealed a two-step curve, in which the first site plateaued before 25% FRET efficiency (*K*_D(High)_ = 0.5 nM), while the second site displayed weaker affinity (*K*_D(Low)_ = 30 nM; Fig. 1e, Supplementary Fig. 2f, and Table 1). Thus, the two SIRT6 binding sites on a nucleosome are asymmetric, which suggests that two separate mechanisms of binding occur to form a tight complex.

### Identification of interacting regions on SIRT6 and NCPs

To map the binding regions and provide residue-level insight into how SIRT6 associates with nucleosomes, we employed a method that incorporates hydrogen-deuterium exchange (HDX) coupled to mass spectrometry^22^. The SIRT6:NCP complex, or the individual SIRT6 and nucleosome constituents, were analyzed by differential HDX to determine regions that are perturbed in the complex. Peptides that exhibit more protection from HDX in the complex might indicate sites of protein–protein interactions, while those that display more exchange may represent regions of increased structural plasticity. The results showed significant HDX changes in both SIRT6 and NCPs indicative of specific binding regions or conformational changes.

Nucleosomes that were bound to SIRT6 experienced more exchange in the H4 C-terminus (residues 87–102), indicating a possible structural destabilization induced by SIRT6 (Supplementary Fig. 3a). The H4 C-terminus is known to be structurally plastic, as recent studies of SNF2H- and HP1-bound NCPs revealed that this octamer region can experience increased solvent exposure due to protein binding^23,24^. Importantly, the H4 residues are buried in the canonical nucleosome structure^16^, which suggests that SIRT6 interactions with other histone domains or nucleosomal DNA perturb the NCP structure.

**Fig. 2:**
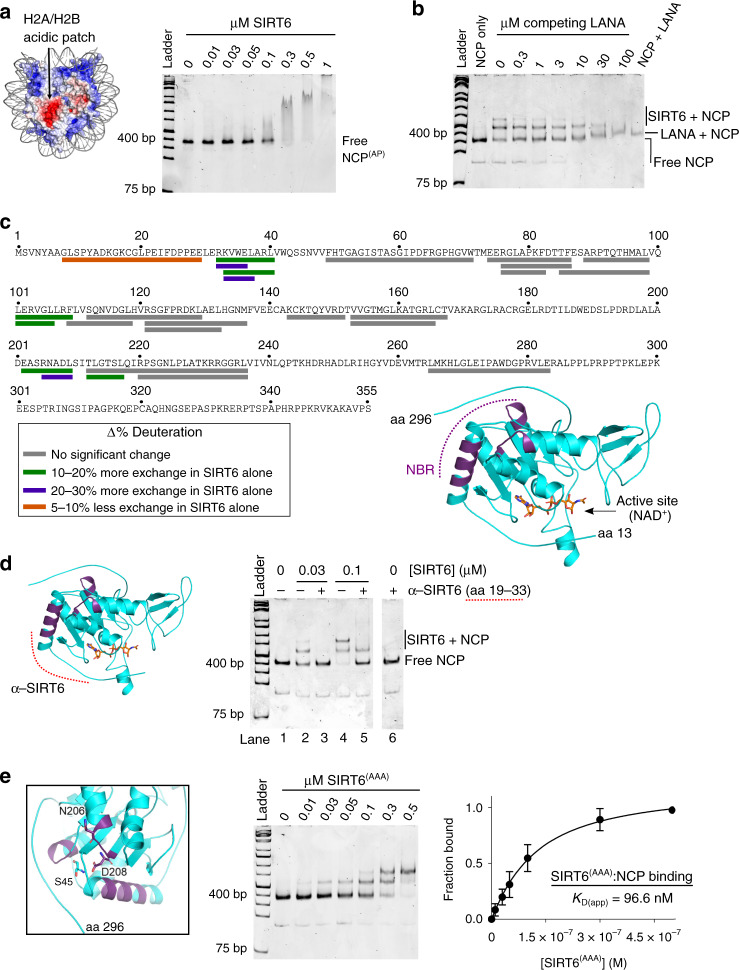
SIRT6 and nucleosomes interact through specific regions. **a** Surface depiction of the nucleosome (3lz0.pdb^64^) with electrostatics generated by the APBS plugin for PyMOL^65,66^. One acidic patch on one side of the NCP is indicated. NCPs assembled with four alanine mutations at the acidic patch (NCP^(AP)^) exhibited weaker binding to SIRT6. The image is representative of three independent experiments. **b** In the LANA peptide competition EMSA, SIRT6:NCP complexes were prepared with 100 nM SIRT6 and 50 mM NCP, then competed with LANA peptide. The image is representative of three independent experiments. **c** HDX-MS analysis of differential peptide exchange in the SIRT6:nucleosome interaction. SIRT6 residues that are protected against exchange when bound to NCPs are highlighted in green and purple colors in the primary sequence, and in purple in the structure of SIRT6 (3pkj.pdb^8^). NAD^+^ (nicotinamide adenine dinucleotide) is included to indicate the active site. **d** An antibody that recognizes SIRT6 amino acids 19–33 (red dashed line) was used to probe SIRT6 binding to NCPs. When pre-bound to SIRT6, the antibody disrupts formation of both the 1:1 and 2:1 SIRT6:nucleosome complexes. The image is representative of three independent experiments. **e** The zoomed image displays amino acids mutated to alanine (SIRT6^(AAA)^) for native gel analysis. The residues occupy loops that reside around a specific interface. On the right, EMSA of SIRT6^(AAA)^ binding to nucleosomes shows a weakened binding affinity. Data are presented as mean ± s.d. from four independent experiments. Source data are provided as a source data file.

One particular structural feature frequently utilized by nucleosome-interacting partners is an H2A/H2B acidic patch, which is located on either side of the NCP disc-like structure (Fig. 2a)^17,25^. To test SIRT6 binding to the acidic patch, we reconstituted nucleosomes bearing four alanine mutations on acidic H2A residues at the patch (NCP^(AP)^; E61, E64, D90, E92). In EMSA experiments, SIRT6 exhibited weaker binding to NCP^(AP)^ than to wild-type nucleosomes (*K*_D(app)_ = 138 nM, Fig. 2a and Supplementary Fig. 3b). Importantly, the bound species observed were slower migrating complexes, in contrast to the specific 1:1 and 2:1 SIRT6:NCP complexes previously observed (Fig. 1a). To further probe SIRT6 binding to the acidic patch, we used the Latency Associated Nuclear Antigen (LANA) peptide from Kaposi’s sarcoma-associated herpesvirus as a competitor of SIRT6:NCP interactions in EMSAs^25^. LANA clearly disrupted the 2:1 SIRT6:NCP complex into a 1:1 complex at the lowest LANA concentration tested (300 nM), and abrogated all binding at 100 µM peptide concentration (IC_50_ = 9.5 µM; Fig. 2b and Supplementary Fig. 3b). Together, these data are consistent with both SIRT6 molecules utilizing the nucleosomal acidic patch. The two SIRT6 proteins likely each occupy a separate acidic patch, given that SIRT6 remains monomeric under the concentrations used, and the acidic patch is not a large interface known to bind multiple proteins simultaneously^17^.

If LANA displaces SIRT6 from nucleosomes, then one would predict that this peptide might disrupt SIRT6 activity on nucleosomes. To assess nucleosome deacetylation, we utilized native chromatin from HCT116 cells as substrate. Cells were treated with trichostatin A to preserve acetylation levels before lysis, then digested with micrococcal nuclease A (MNase A) to generate mono- and poly-nucleosomes. In the presence of LANA peptide, SIRT6 activity on HCT116 nucleosomes was decreased compared to control reactions without peptide (Supplementary Fig. 3c). Thus, binding through the acidic patch supports SIRT6 function on nucleosomes.

On the SIRT6 structure, nucleosome binding increased hydrogen-deuterium exchange at the N-terminal loop (Fig. 2c; residues 8–29). Mutations of candidate charged residues in this loop did not change the affinity for nucleosome binding (Supplementary Fig. 1d). Thus, this N-terminal loop does not appear to contribute binding energy. Within nearby structural regions, we observed protection from exchange in the N-terminal helix, as well as in peptides corresponding to residues 101–108 and 202–209. Notably, all residues that experienced significant protection cluster to a common interface that is adjacent to the C-terminus and distant from the active site (Fig. 2c). Given the significant HDX changes in this interface, we hypothesized that it represents a nucleosome-binding region (NBR).

To determine if the N-terminal helix (residues 28–43) in the NBR participates in NCP interactions, we employed an antibody raised against SIRT6 residues 19–33 (Fig. 2d). SIRT6 can form a 1:1 complex exclusively in native gels at 30 nM protein concentration, or form both 1:1 and 2:1 SIRT6:NCP complexes at 100 nM SIRT6 (Fig. 2d; lanes 2 and 4). Thus, the antibody was allowed to associate with SIRT6 before NCP binding. The complexes were then monitored in EMSAs, where disassembly or supershifting might occur, depending on whether the epitope was essential or excluded in SIRT6:NCP binding, respectively. The results showed that the antibody fully disrupted assembly of both the 1:1 and 2:1 SIRT6:NCP complexes (lanes 3 and 5). Thus, these data support the N-terminal helix engaging with both the high- and low-affinity binding sites on NCPs.

In addition to antibody competition, we introduced strategic amino acid mutations in SIRT6. A set of mutations made within the N-terminal helix (34A/35A/37A) was not bacterially expressed, while those that included amino acids 101–108 completely eliminated enzyme activity (Supplementary Fig. 1c), making these mutants unsuitable for validation. Simultaneous alanine substitutions at three SIRT6 residues, however, retained protein structure and function (S45, N206, and N208 (SIRT6^(AAA)^); Supplementary Fig. 1a–c). S45 was not represented among peptides identified from HDX analysis, but is positioned in a loop immediately following the N-terminal helix, in close proximity to the loop occupied by N206 and N208 (Fig. 2e). SIRT6^(AAA)^ displayed a 7-fold weaker *K*_D_ for NCPs in EMSAs (*K*_D(app)_ = 96.6 nM; Fig. 2e and Table 1). The moderate change in affinity suggests that the HDX changes in the NBR loops represent either an indirect structural change, or a weak binding contribution that does not supply direct hydrogen bonding.

If the mutations in SIRT6^(AAA)^ are distant from the active site and partially impair nucleosome binding, then we predicted the mutant to retain full activity on peptide substrate while deacetylating nucleosomes with lower efficiency. Wild-type and SIRT6^(AAA)^ proteins exhibited similar rates of deacetylation on H3K9ac peptide, as predicted (Supplementary Fig. 1c). When provided with native HCT116 nucleosomes as substrate, however, SIRT6^(AAA)^ deacetylated H3K9ac at a slower rate compared with wild-type SIRT6 (Supplementary Fig. 3d). Therefore, direct and high affinity binding to nucleosomes, mediated in part by NBR residues S45, N206, and N208, is a requirement for efficient deacetylation.

### The CTD of SIRT6 is required for high-affinity binding

**Fig. 3:**
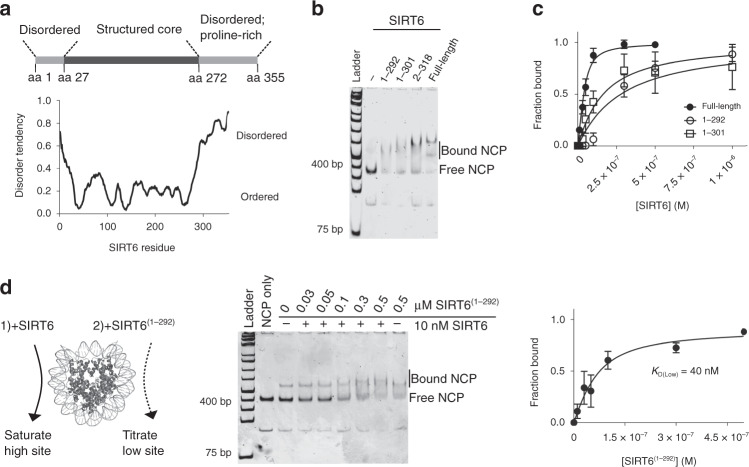
The C-terminus of SIRT6 stabilizes the high-affinity binding site. **a** Schematic of known SIRT6 domains accompanied with a bioinformatic prediction of protein disorder (MetaPrDos) below. The C-terminal domain (CTD) starts at residue 272 and is predicted to be unstructured. **b** EMSA comparison of NCP binding between 100 nM SIRT6 and 500 nM C-terminally truncated SIRT6 variants. The 1:1 SIRT6:nucleosome complex is undetectable in native gels when the CTD is truncated, even at lower protein concentrations (Supplementary Fig. 4b). The image is representative of three independent experiments. **c** As a result, SIRT6^(1–292)^ and SIRT6^(1–301)^ associates with nucleosomes with an order of magnitude weaker affinity. The *K*_D_s were calculated with a ligand-depleted equation from titration of SIRT6^(1–292)^ or SIRT6^(1–301)^ to 50 nM NCP. Data are presented as mean ± s.d. from three independent experiments. **d** The weaker binding site on nucleosomes (*K*_D(Low)_) does not require the CTD. The high affinity site on nucleosomes was saturated with 10 nM SIRT6, then increasing concentrations of SIRT6^(1–292)^ was added to bind the available site. Although the formation of the fully formed 2:1 complex is still apparent, it migrates too close to the 1:1 band to accurately quantify. Thus, fraction bound was instead calculated by subtracting the fraction of free nucleosomes still present. Data are presented as mean ± s.d. from three independent experiments. Source data are provided as a source data file.

The HDX results suggested that a specific SIRT6 interface associates with nucleosomes, yet the enzyme engages through asymmetric binding modes, utilizing two sites with different affinities. This implies that a separate SIRT6 domain contributes to the favorable binding energetics at the high affinity site. Residues at the disordered, highly basic C-terminal domain (CTD), which spans the final 83 amino acids and shares little sequence identity with other nuclear sirtuins, were not represented in any peptide identified in HDX analysis (Figs. 2c and 3a and Supplementary Fig. 4a). Thus, we introduced truncations of varying lengths at the C-terminus and evaluated these variants for nucleosome binding (Fig. 3b). The C-terminal deletion mutants exclusively formed 2:1 SIRT6:NCP complexes in the native gel without a detectable 1:1 intermediate, suggesting that the high affinity site was compromised, which led to a similar affinity for both binding events. As a result, the overall *K*_D_ of SIRT6^(1–292)^ and SIRT6^(1–301)^ (*K*_D_ = 254 and 139 nM, respectively) to nucleosomes is an order of magnitude weaker than full-length SIRT6 (Fig. 3c, Supplementary Fig. 4b, and Table 1). Thus, the C-terminally truncated mutants display impaired binding to NCPs, as well as a loss of the asymmetric binding mechanism.

Given these changes in binding with the SIRT6^(1–292)^ and SIRT6^(1–301)^ mutants, we reason that the CTD is exclusively utilized at the high affinity site. Under this model, binding to the lower affinity site (*K*_D(Low)_) would be similar between SIRT6 and SIRT6^(1–292)^. To test this mechanism, we first saturated the high affinity site on NCPs with 10 nM full-length SIRT6, then added increasing concentrations of SIRT6^(1–292)^ and subjected the resulting complexes to native gel analysis (Fig. 3d and Table 1). The data revealed a dissociation constant (40 nM) similar to the *K*_D(Low)_ value reported by the FRET experiment (Fig. 1e), supporting a model in which the CTD contributes to only the higher affinity binding event.

To further interrogate this mechanism, we used an antibody raised against SIRT6 C-terminal residues 330–348 as a probe in EMSA experiments. The antibody was allowed to bind full-length SIRT6 before nucleosome addition (Supplementary Fig. 4c). When only the 1:1 SIRT6:NCP complex is apparent at 20 nM SIRT6, the antibody fully disrupted this complex (lanes 3 and 4). When both the 1:1 and 2:1 complexes appeared at 100 nM SIRT6, the antibody displaced part of the bound population while supershifting the rest (lanes 5 and 6). Finally, when both binding sites were saturated, only supershifting occurred, likely due to CTD-independent interactions driving binding at higher SIRT6 concentrations (lanes 8 and 9). Collectively, the results are consistent with the observation that the CTD is not necessary for complete SIRT6:NCP assembly (Fig. 3b), yet is essential for tight and multivalent binding at a single, high affinity site.

### The CTD is an intrinsically disordered DNA-binding domain

**Fig. 4:**
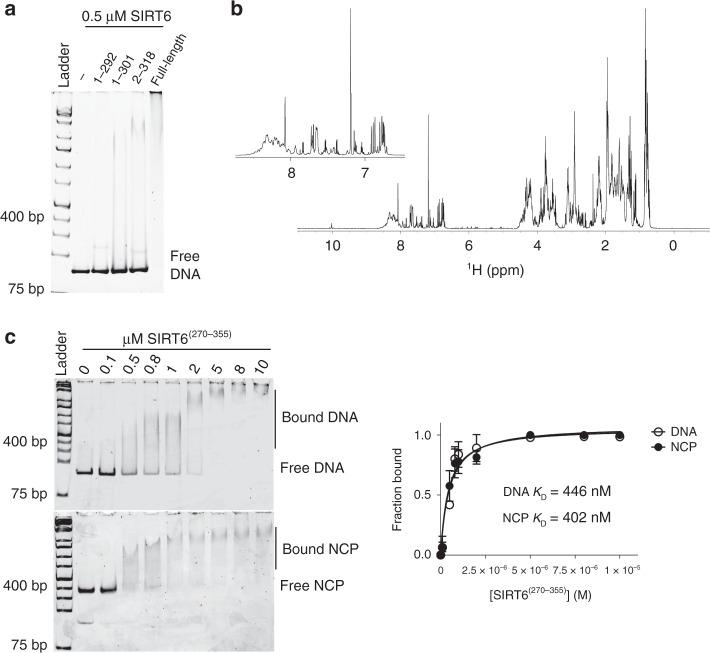
The C-terminal domain of SIRT6 is an intrinsically disordered DNA-binding domain. **a** EMSA of SIRT6 and SIRT6 C-terminal deletion mutants bound to 601 sequence DNA reveals that full-length SIRT6 has highest affinity for DNA. All SIRT6 proteins were used at 500 nM. The image is representative of three independent experiments. **b** ^1^H NMR spectrum of recombinant CTD (residues 270–355) shows a cluster of peaks at ~7.0–8.5 ppm. The inset is a zoom of the spectrum at ~6.5–8.5 ppm. **c** EMSA of increasing concentrations of CTD bound to 50 nM DNA (top gel) and nucleosomes (bottom gel). The data was fitted to a ligand-depleted equation and shows superimposing binding isotherms. Data are presented as mean ± s.d. from three independent experiments. Source data are provided as a source data file.

The CTD establishes tighter binding on NCPs, suggesting that this unique domain recognizes additional structural feature(s) on the nucleosome. To test if it is necessary to bind the acidic patch, we used the LANA peptide to measure inhibition of SIRT6^(1–292)^:NCP binding (Supplementary Fig. 5a). The LANA peptide displaced the interaction between SIRT6^(1–292)^ and NCPs with a similar IC_50_ (6.1 µM) as observed for the full-length SIRT6:NCP complex (Supplementary Fig. 3b and Table 1), indicating that the folded core binds the acidic patch while the CTD engages other parts of the nucleosome. Given the presence of many basic residues along the CTD (pI = 10.4) and the propensity of proline-rich domains to function as DNA-binding regions^26,27^ (Supplementary Fig. 4a), we investigated several SIRT6 C-terminal deletion mutants for DNA binding. In EMSA experiments using 500 nM protein incubated with the 601-positioning DNA sequence, full-length SIRT6 displayed highest affinity for DNA, while the mutants bound noticeably weaker (Fig. 4a). In addition, the NBR mutant SIRT6^(AAA)^ fully bound DNA under the same conditions, consistent with the folded core and the CTD of SIRT6 having distinct binding contacts (Supplementary Fig. 5b).

To confirm that the CTD directly interacts with DNA, we expressed and purified the isolated domain (residues 270–355) and evaluated DNA binding capacity (Supplementary Fig. 1a). The domain has no known ordered structure: the only CTD residues observed in the SIRT6 crystal structure, 272–296, form a disordered loop^8^, while bioinformatic predictions indicate that the entire domain is intrinsically disordered (Fig. 3a). To confirm these predictions, we determined the ^1^H NMR spectrum of the CTD and observed peak clustering at ~7.0–8.5 ppm, which is characteristic of intrinsically disordered domains (Fig. 4b). In EMSA experiments, we found that the CTD bound random sequences of DNA in a commercial 10 base pair ladder, as well as the 601-positioning sequence (Fig. 4c and Supplementary Fig. 5c). More strikingly, binding to the latter sequence with and without the histone octamer assembled on it produced exact binding isotherms. This reveals that the CTD is not only indiscriminate of DNA sequences, but recognizes both linear DNA as well as the curved, distorted DNA inherent in nucleosomes. Moreover, the LANA peptide did not disrupt this CTD:nucleosome interaction (Supplementary Fig. 5d). Thus, the SIRT6 CTD is a unique module among sirtuins that aids in nucleosome engagement solely through a DNA-dependent mechanism.

The observation that SIRT6 can bind to both nucleosomal DNA and the acidic patch suggests that the overall complex is governed by multivalent interactions. To provide additional support, we tested if specific complexes can form when SIRT6^(1–292)^ or SIRT6^(1–301)^ is incubated with NCP^(AP)^. Under these conditions, without available CTD-dependent DNA interactions and acidic patch docking, no specific binding was observed up to 1 µM (Supplementary Fig. 5e). Thus, multiple contact points are involved in stabilizing the SIRT6:NCP complex.

### The CTD promotes efficient chromatin deacetylation by SIRT6

**Fig. 5:**
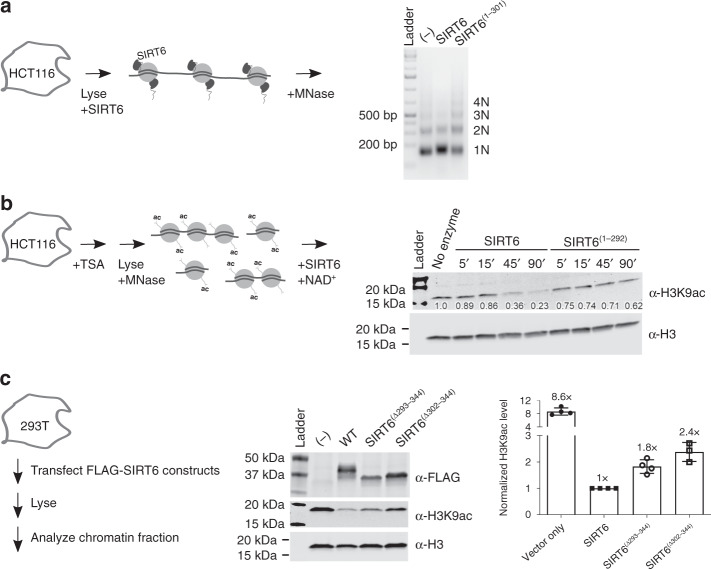
SIRT6 is a more efficient enzyme when bound to endogenous chromatin through the CTD. **a** MNase digestion of HCT116 chromatin in the presence of SIRT6 or SIRT6^(1–301)^ reveals that the CTD contributes to greater sensitivity of native chromatin to digestion. 10 µM SIRT6 or SIRT6^(1–301)^ (or buffer as negative control) was incubated with lysed HCT116 cells for 1 h before 5 min of MNase treatment. Equivalent DNA content among samples was loaded in an agarose gel. The image is representative of three independent experiments. **b** 100 nM SIRT6 or SIRT6^(1–292)^ was monitored for H3K9ac deacetylase activity on native HCT116 nucleosomes by immunoblotting. The numbers below the bands indicate the H3K9ac level relative to the no enzyme control, all normalized to total H3 signal. **c** 293T cells transfected with empty FLAG tag vector, FLAG-tagged SIRT6, or the indicated FLAG-tagged mutants were assessed for H3K9ac levels. After 48 h, the chromatin fraction was separated and blotted for H3K9ac. Deletions within the CTD result in ~2x more acetylation. Data are presented as mean ± s.d. from three independent experiments. Source data are provided as a source data file.

The tight binding imparted through the CTD may provide the full-length enzyme with a thermodynamic advantage to interact with chromatin substrates. Thus, to determine if the CTD-dependent binding mechanism applies to native chromatin, we incubated SIRT6 or SIRT6^(1–301)^ with chromatin from lysed HCT116 cells, then subjected the cells to MNase digestion. In the presence of full-length SIRT6, a substantial fraction of chromatin was strictly digested into mono-nucleosomes compared to negative control, whereas SIRT6^(1–301)^-associated chromatin did not exhibit an altered digestion pattern (Fig. 5a). Thus, the CTD on full-length SIRT6 increased the nuclease sensitivity of poly-nucleosomes, which suggests that the domain imparts SIRT6 with direct, high-affinity binding with native chromatin.

To test if enhanced binding through the CTD also promotes more efficient histone H3K9ac deacetylation, we investigated the ability of full-length SIRT6 or various truncated mutants to deacetylate native nucleosomes. Our binding studies above predict that full-length SIRT6 would be a more efficient enzyme than a counterpart without the CTD. Indeed, when provided with MNase-digested nucleosomes as substrate, the full-length enzyme efficiently deacetylated endogenous H3K9ac, while SIRT6^(1–292)^ and SIRT6^(1–301)^ deacetylated the modification much slower (Fig. 5b and Supplementary Fig. 6a). Therefore, efficient chromatin deacetylation requires the CTD-dependent nucleosome-binding mechanism.

To corroborate the contribution of the CTD to chromatin binding and enzyme activity in a cellular environment, we examined the localization of SIRT6 C-terminal deletion mutants, as well as their ability to deacetylate chromatin (H3K9ac levels), in cultured cells transiently overexpressing the constructs. Because the nuclear localization motif is among the last 11 amino acids, these residues were retained in the truncated proteins (SIRT6^Δ293–344^ and SIRT6^Δ302–344^)^19^. To analyze the sub-cellular distribution of the SIRT6 constructs, we fractionated HCT116 cells into cytoplasmic and nucleoplasmic fractions. Compared to wild-type SIRT6, SIRT6^Δ293–344^ and SIRT6^Δ302–344^ levels were higher in both fractions (Supplementary Fig. 6b), suggesting that truncating the CTD led to weakened chromatin association. Next, we assessed the relative deacetylation of H3K9ac in HEK 293T cells expressing wild-type or truncated SIRT6. Cells overexpressing the full-length enzyme showed dramatically lowered H3K9ac levels, as predicted from our in vitro assays and consistent with prior reports^7,19^ (Fig. 5b, c). Acetylation, however, was markedly higher in cells overexpressing the mutants (Fig. 5c). These phenotypes are consistent with the biophysical experiments conducted above, as truncated SIRT6 retains catalytic activity, yet lacks the CTD-driven binding on nucleosomal substrates. Thus, direct and high-affinity binding to nucleosomes drives efficient substrate deacetylation, a characteristic of SIRT6 unique among deacetylases.

## Discussion

Multiple animal studies indicate critical cellular functions of SIRT6 that promote longevity through regulation of metabolism and genome maintenance^3–6^. SIRT6 associates with chromatin and can dramatically decrease global levels of H3 acetylation, but it was unclear how SIRT6 can transform bulk chromatin. Does SIRT6 possess the inherent capacity to tightly bind nucleosomes and perform efficient deacetylation? Here, we reveal that the assembly of a high affinity SIRT6:nucleosome complex is functionally self-contained to execute efficient H3 deacetylation.

This tight interaction is rare among chromatin-modifying enzymes, as only the Set8 methyltransferase has a similar affinity for nucleosomes^28^. Importantly, many histone deacetylases exist in multi-subunit complexes that rely on other complex members for nucleosome targeting^18^. The best characterized examples include HDAC1 and HDAC2, which are both part of the Sin3, NuRD, and coREST complexes, ensembles that all have additional nucleosome-binding subunits^29–35^. To our knowledge, SIRT6 is the only histone deacetylase to associate tightly with nucleosomes through a single polypeptide. We note that this high affinity interaction is well aligned with ascribed cellular functions. Overpressed SIRT6 is largely observed in the chromatin fraction of nuclei^3,19,36^, consistent with our own observations (Supplementary Fig. 6b). As SIRT6 activity is important for cellular homeostasis, having a pool of tight-binding enzyme would be beneficial for prompt deacetylation of histones. Indeed, chromatin occupancy of SIRT6 is enhanced during DNA damage and disturbed by oncogenic mutations, revealing that localization regulates function^36,37^. Moreover, given the highly charged nature of SIRT6 (full-length pI = 9.5) and the presence of a long disordered domain, tight residence on chromatin may be a mechanism to protect SIRT6 stability.

Despite reports that SIRT6 is recruited to chromatin through interactions with NF-ΚB, Lamin A, and nucleosomes^20,38,39^, the current study demonstrates that SIRT6 can engage nucleosomes with low nanomolar affinities, which would appear sufficient to place SIRT6 on chromatin without the need for additional binding mechanisms. Instead, interactions with other proteins are likely regulatory of SIRT6 activity. For instance, these proteins might restrict or inhibit SIRT6 binding/activity, or alternatively, shepherd SIRT6 to specific gene promoters. Furthermore, many other proteins and peptides can also occupy the H2A/H2B acidic patch and/or nucleosomal DNA, which might provide obstacles for SIRT6:NCP interactions^17^. SIRT6 occupancy could also be tuned by NCP or SIRT6 post-translational modifications that alter the equilibrium of the complexes. Together, our study provides a simple mechanism for how a single polypeptide chain can perform chromatin deacetylation at the global level.

**Fig. 6:**
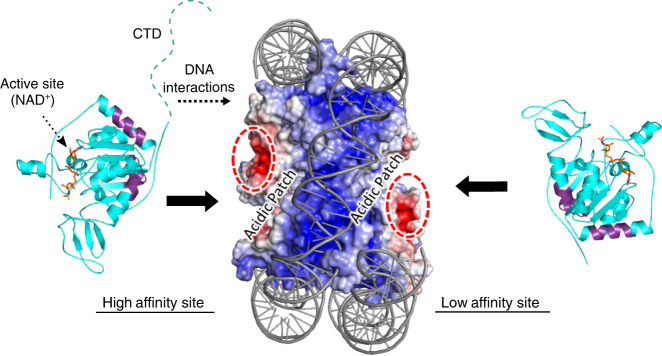
Multivalent interactions comprise the SIRT6:nucleosome interaction. The nucleosome core particle is shown in surface representation and the SIRT6 structure (residues 13–296) is in cartoon. Under the simplest model, each acidic patch on either side of the nucleosome supports binding to one SIRT6 molecule, depicted here with purple residues representing the most protected peptides in HDX analysis (Fig. 2c). The CTD is necessary for interactions with nucleosomal DNA at the higher affinity site.

Along this vein, SIRT6 engages a nucleosome core particle with multiple regions, including a binding interface (NBR) that comprises the N-terminal helix and an adjacent interface, as well as the disordered CTD (Fig. 6). In a 2:1 SIRT6:nucleosome arrangement, both bound SIRT6 molecules likely employ the NBR-dependent mechanism, as revealed by N-terminal antibody competition experiments, while the CTD is exclusive for the high affinity site only (Figs. 2d and 3d). Given the proximity of the NBR to the CTD, we speculate that CTD-driven binding may be a means to efficiently escort the structured core of SIRT6 to the nucleosome. Intrinsically disordered, charged domains are a common characteristic of chromatin-binding proteins, which use such domains to enable fast association with target binding partners, steering the interaction toward specific contacts with the folded protein core^40–43^. Because the CTD is unique to SIRT6, as no other nuclear sirtuin possesses such a domain similar in sequence and charge (Supplementary Fig. 4a), we speculate that this CTD-dependent mechanism is also a distinguishing feature of SIRT6. We propose that the nucleosomal DNA-binding function of the CTD allows the protein to rapidly find nucleosomes within nuclear confines. Further studies investigating the on and off rates driven by the CTD in vitro and in cells would determine if this is a plausible mechanism.

Interestingly, only a single SIRT6 molecule employs the CTD for binding a nucleosome core particle, suggesting that the CTD is specific for a single nucleosomal DNA region. This mechanism would be consistent with the many nucleosome-binding proteins that have preference for specific regions on nucleosomal DNA, which is inherently asymmetric and features gyres of varying curvatures^17,44–46^. Although the isolated CTD exhibits promiscuous DNA-binding ability, the specific binding displayed when engaging NCPs suggests that the CTD is sterically restricted as part of the full-length SIRT6 protein, preventing non-specific DNA binding (Fig. 1a).

The unbound CTD, consequently, may be accessible for potential protein–protein or protein–DNA interactions. Given the links between SIRT6 activity and DNA damage responses, transcription, and telomere stability, the available CTD may recruit other factors critical for these processes^1^. Indeed, the CTD is required for SNF2H and CHD4 recruitment to damaged DNA for efficient nucleosome remodeling^13,14^. Furthermore, as SIRT6 rapidly localizes to sites of DNA damage, the CTD may play a role in binding damaged DNA or initiating proper cellular responses to DNA damage. In support, a recent report reveals that ectopic GFP-SIRT6 localizes to damaged DNA independent of other known DNA repair proteins^47^. Mutagenesis of predicted DNA-binding residues in the folded core of SIRT6 disrupted in vitro DNA binding to a minor extent, albeit at unknown protein concentrations and with unknown protein stability^47^. Although we do not observe SIRT6:DNA binding without the CTD at 500 nM protein concentration (Fig. 4a), there may be multiple electrostatic contact points for DNA evident at higher SIRT6 concentrations. The impact of relevant mutations – at the CTD or at other putative sites – on SIRT6 recruitment to damaged DNA would determine which site(s) are essential for DNA damage responses. Taken together, the free CTD may be a module that enables SIRT6 to function in concert with multiple pathways.

On the nucleosome structure, SIRT6 molecules occupy the acidic patches formed by H2A/H2B residues (Fig. 2a, b). Although we do not observe differences in HDX in histone peptides corresponding to the acidic patch residues, the lack of coverage for H2A and the long peptide lengths seen in H2B may preclude such changes (Supplementary Fig. 3a). Surprisingly, the positively charged CTD does not associate with the acidic patch, revealing that it has a strict and strong preference for DNA (Supplementary Fig. 5d). The folded core of SIRT6, therefore, participates in the acidic patch interaction. Because the LANA peptide disrupts binding of both SIRT6 molecules on the NCP, the simplest model is that the two SIRT6 molecules each occupy a separate acidic patch on either side of the nucleosome (Fig. 6). This mechanism is consistent with SIRT6 remaining monomeric, and with the small surface area available on the acidic patch (Fig. 1c)^17^. However, high resolution structural studies are needed to ascertain which SIRT6 residues are directly involved, and how the SIRT6 conformation might change. For example, recent structural studies reveal that the Dot1L methyltransferase also uses the acidic patch as part of a nucleosome-binding mechanism, an interaction that restricts Dot1L to the proper orientation needed to efficiently methylate H3K79^48–51^. Thus, whether the SIRT6 conformation on nucleosomes experiences a similar change awaits further structural analysis.

Aside from the acidic patch, we also observed destabilization (increased exchange) at the H4 C-terminus (Supplementary Fig. 3a). This H4 domain is noted to have structural plasticity: it adopts a parallel β-sheet to interact with nucleosomal H2A, yet rotates almost 180° to form an anti-parallel sheet with the histone chaperone Asf1^16,52^. Partial deletion of the H4 C-terminus leads to a destabilized nucleosome structure, while modifications near this domain lead to increased nucleosome unwrapping, consistent with a site that undergoes rapid DNA breathing^17,53,54^. Because the H4 C-terminal residues are buried in the nucleosome, the simplest explanation for SIRT6-induced destabilization is that SIRT6 interactions with histones and/or DNA perturb the nucleosome structure. In support, recent studies show that other proteins, including HP1 and the acidic patch-binding SNF2H, also increase octamer plasticity at the H4 C-terminus, revealing that even buried histone residues are susceptible to conformational changes as a result of protein-nucleosome binding^23,24^. Future work is needed to determine if the SIRT6-induced H4 changes have functional consequences on chromatin structure.

The mechanisms uncovered in this work reveal how an enzyme engages its cognate substrate, a complex structure with multiple possible docking sites. The assembly of a high affinity SIRT6:NCP complex allows SIRT6 to leverage the favorable free energy changes from nucleosome binding to support thermodynamic benefits toward deacetylation. In light of the many ongoing efforts to develop small molecule effectors of SIRT6^55–57^, these unique mechanisms illuminate previously unknown roles of SIRT6 domains, and reveal that binding and activity in the context of nucleosomes is an important consideration.

## Methods

### Protein and nucleosome preparation

Recombinant nucleosomes with 601-positioning 147-bp DNA and x. laevis histones were reconstituted using the salt gradient dialysis method, in which equimolar histone octamers and DNA were slowly dialyzed from 2 M NaCl to 10 mM NaCl^58^. Histones H2A, H2B, H3, and H4 were individually expressed in BL21(DE3) competent cells, then purified from inclusion bodies under denaturing conditions by ion exchange chromatography. To refold octamers, the histones were combined in equimolar ratios and resolved through size exclusion chromatography. 601-positioning DNA was generated from Eco32I digest from 32-mer inserts in a pUC19 vector (a generous gift from Peter W. Lewis)^21^.

To prepare Cy3-labeled nucleosomes, Cy3 was positioned on the first nucleotide of the 147-bp 601 sequence using the following primers from Integrated DNA Technologies:

Forward primer: /5Cy3/CTGGAGAATCCCGGTGCCG

Reverse primer: ACAGGATGTATATATCTGACACGTGCCTGG

The 601 sequence was PCR-amplified with the primers, then cleaned using silica columns from the Qiagen PCR Purification kit. Nucleosomes were assembled with octamers and the Cy3-labeled DNA using salt gradient dialysis. The degree of labeling was typically >97%.

SIRT6 mutants were introduced using site-directed mutagenesis using overlapping primers bearing the desired mutation. The mutations either introduced alanine for the SIRT6^(AAA)^ mutant or a stop codon to generate SIRT6^(1–292)^ and SIRT6^(1–301)^. All mutants were amplified with Phusion polymerase (Thermo Fisher) and sequence verified.

His-tagged SIRT6 and SIRT6 mutants in a pQE80 vector were overexpressed in BL21(DE3) competent cells by induction with 0.5 mM IPTG at an OD_600_ of 0.8 for 18 h at 25 °C^8^. After harvest, the cells were sonicated and the lysate resolved by nickel chromatography. The proteins were further purified through a Heparin column, and finally dialyzed in 50 mM Tris, 150 mM NaCl, 0.1 mM TCEP, 5% glycerol, and pH 7.4. The CTD (residues 270–355) was purified using the same methods, except the CTD was dialyzed in 20 mM sodium phosphate, 100 mM NaCl, and pH 7.3.

For fluorophore labeling on SIRT6, only two cysteines are available for maleimide-based conjugation (cysteines 18 and 320), as the other cysteines in the sequence are occupied with zinc coordination. To enable site-specific installation, we mutated either C18 or C320 to serine, leaving the other available. When both are mutated, negligible labeling was detected. For TAMRA maleimide (Anaspec) labeling at residue 320, a C18S mutant was purified, then incubated with 10x molar excess dye for 16 h at 4 °C. The mixture was then run through a homemade column with G25 sephadex resin (GE Healthcare) to remove unbound dye. The absorbance profile indicated 1:1 molar labeling. For TQ3 maleimide (AAT Bioquest) labeling, the dye was positioned on C18, given the weaker apparent affinity observed in EMSAs for the SIRT6^(TAMRA)^ protein conjugated at the C-terminus, along with subsequent observations that the C-terminus is important for binding NCPs (Figs. 1b and 3b, c). The same procedure was followed, resulting in 80% labeling efficiency. Pyrene (Sigma) was also labeled at C18 with 60% efficiency.

### Electrophoretic mobility shift assays

For nucleosome-binding assays, 50 nM of recombinant nucleosomes were incubated in assay buffer (10 mM Tris, 50 mM NaCl, 0.5 mM TCEP, 6% glycerol, and pH 7.4) with varying SIRT6 concentrations in 7 µL reactions. The reactions were performed on ice for >15 min in siliconized or LoBind tubes (Eppendorf) to minimize protein adherence to material. The reactions were then resolved in a 0.2x TBE 5% 59:1 acrylamide:bis-acrylamide gel for 90 min at 70 volts on ice. A DNA ladder (GeneRuler 1 kb Plus; Thermo Fisher) was typically included in the gels. The gel was stained with SYBR Safe (Thermo Fisher) and imaged on a Typhoon 9000 imager (GE Healthcare) using Typhoon Control Software (v1.1). SYBR Safe fluorescence was excited at 473 nm and scanned with a 510 nm filter cutoff, while TAMRA was excited at 532 nm and read using a 575 nm filter. For DNA-binding assays with 147 bp 601 sequence DNA, the same conditions applied, except 100 mM NaCl was used in the buffer. To test CTD binding to a DNA ladder, 500 nM of a 10 step ladder (Promega) was bound with varying concentrations of protein. Each assay was repeated at least three times independently.

The bands were quantified with ImageJ (v1.52d): fraction bound was determined by calculating the integrated densitometry values (IDVs) of the unbound nucleosome band (bottom) and the shifted complex(es) as the bound fraction.

1 $${Y} = ({\mathrm{IDV}}_{{\mathrm{Bound}}} - {\mathrm{IDV}}_{{\mathrm{Bound}}\,{\mathrm{Background}}}) \,	\div ({\mathrm{IDV}}_{{\mathrm{Bound}}} - {\mathrm{IDV}}_{{\mathrm{Bound}}\,{\mathrm{Background}}}\\ \, 	+ {\mathrm{IDV}}_{{\mathrm{Free}}} - {\mathrm{IDV}}_{{\mathrm{Free}}\,{\mathrm{Background}}}),$$

where *Y* is fraction bound.

To fit the isotherm, a quadratic equation was used in Prism 8 (GraphPad) to account for ligand depletion:

2 $${Y} = {\mathrm{Bmax}}\, \times ((({K}_{\mathrm{D}} + {A} + {X}) - {\mathrm{sqrt}}(((K_{\mathrm{D}} + {A} + {X})^ \wedge 2) - (4 \times {A} \times {X}))) \div (2 \times {A})),$$

where *A* is the concentration of nucleosomes (50 nM), Bmax is maximum binding, and *X* is the varying concentrations of protein titrated into A.

In competition experiments with N- and C-terminal specific antibodies (Abcam ab62739 and ab62738, respectively), the antibody was allowed to pre-bind SIRT6 in the reaction buffer before nucleosomes were added. The final concentrations of the N- and C-terminal specific antibodies were 0.04 mg/mL and 0.15 mg/mL, respectively.

In experiments using LANA peptide (MAPPGMRLRSGRSTGAPLTRGSC; GenScript), SIRT6 was allowed to bind nucleosomes first, followed by LANA competition. Fraction bound was calculated according to Eq. (1) and then converted to percent inhibition using Eq. (3). IC_50_ values were then calculated by Eq. (4) from fitting in Prism 8.

3 $${\mathrm{Percent}}\;{\mathrm{inhibition}} = 100 - \left( {100 \times {Y}} \right),$$

where *Y* is fraction bound.

4 $${\mathrm{Percent}}\;{\mathrm{inhibition}} = 100 \times I \div ({\mathrm{IC}}_{50} + I) ,$$

where *I* is the concentration of LANA peptide.

### Fluorescence spectroscopy

All FRET experiments were conducted in a Quanta Master 400 fluorometer (Horiba) in assay buffer consisting of 50 mM Tris, 50 mM NaCl, 0.5 mM TCEP, and pH 7.5. For each experiment, 1 nM of Cy3-labeled nucleosomes were equilibrated in a 500-µL quartz cuvette (Starna), then titrated with SIRT6^(TQ3)^. Cy3 was excited at 545 nm (slit width: 5 nm) and the emission was recorded from 560 to 700 nm (slit width: 9 nm) using FelixGX software (v4.0). The titration was repeated four times independently.

Because TQ3 has minimal fluorescence, FRET efficiency was calculated by measuring the decrease in donor intensity:

5 $${E} = 1 - ({F} \div {\mathrm{Fi}}),$$

where *E* is FRET efficiency, *F* is Cy3 emission when bound to a given SIRT6 concentration, and Fi is the Cy3 emission when unbound.

To calculate the Förster radius, the spectral overlap intergral, *J*, was computed from the spectra of Cy3 fluorescence and TQ3 absorbance (Supplementary Fig. 2d).

6 $$R_{\mathrm{o}} = 0.2108 \times {\left( {(\kappa ^2) \times ({n}^ {\mathrm{- 4}}) \times {\mathrm{{\Phi}}} \times {J}} \right)} ^{\mathrm{(1/6)}},$$

where *R*_o_ is the Förster radius, *κ*^2^ is the dipole orientation factor (2/3), *n* is the refractive index of the medium (1.4), and *Φ* is the quantum yield of Cy3, reported to be 0.16 on the 5′-end of duplex DNA^59^.

The Cy3-TQ3 distance was calculated by:

7 $${R} = R_{\mathrm{o}}\times ((1 - {E}) \div {E}) ^ {\mathrm{(1/6)}},$$

where *R* is the distance between donor and acceptor. This leads to a calculated distance of 36 Å. We note, however, that because C18 is located on a flexible loop and two SIRT6^(TQ3)^ molecules are available as acceptors on a nucleosome, this distance is an estimate.

To calculate *K*_D(High)_, the titration points between 0 and 5 nM SIRT6^(TQ3)^ were fitted to Eq. (2) in Prism 8 to account for ligand depletion. To calculate *K*_D(Low)_, the isotherm between 5 nM to 500 nM SIRT6^(TQ3)^ was fitted to a specific binding equation (Eq. (8)) in Prism 8.

8 $${Y} = {\mathrm{Bmax}} \times {X} \div ({K}_{{\mathrm{D}}({\mathrm{Low}})} + {X}).$$

For fluorescence anisotropy experiments, 100 nM SIRT6^(Py)^ was titrated with unlabeled SIRT6 in 20 mM Tris, 150 mM KCl, 2 mM MgCl_2_, 0.5 mM TCEP, 1% glycerol, and pH 7.5. Pyrene was excited at 347 nm (slit width: 5 nm) by light polarized at the vertical and horizontal planes, while vertical and horizontal emission was detected at 378 nm (slit width: 7 nm). The titration was repeated three times independently. Anisotropy was calculated according to Eq. (9).

9 $${r} = ({I_{{\mathrm{VV}}}} - {G} \times {I}_{{\mathrm{VH}}}) \div ({I}_{{\mathrm{VV}}} + 2{G} \times {I}_{{\mathrm{VH}}}),$$

where *I*_VV_ and *I*_VH_ is the intensity of vertically polarized light emitting at the vertical and horizontal planes, respectively, and *G* is the grating factor (*G* = *I*_HV_/*I*_HH_) used to correct for fluorometer sensitivity to polarization bias. The data was fitted to Eq. (8) in Prism 8.

### Hydrogen/deuterium exchange and mass spectrometry

Differential HDX-MS experiments were conducted as follows^22^.

*Peptide identification*: peptides were identified using tandem MS (MS/MS) with an Orbitrap mass spectrometer (Q Exactive, ThermoFisher). Product ion spectra were acquired in data-dependent mode with the top five most abundant ions selected for the product ion analysis per scan event. The MS/MS data files were submitted to Mascot (v2.3.01; Matrix Science) for peptide identification. Peptides included in the HDX analysis peptide set had a MASCOT score >20 and the MS/MS spectra were verified by manual inspection. The MASCOT search was repeated against a decoy (reverse) sequence and ambiguous identifications were ruled out and not included in the HDX peptide set.

*HDX-MS analysis*: SIRT6 alone (5 µM), nucleosomes alone (5 µM), or the SIRT6:nucleosome complex (5 µM) were dialyzed in 10 mM Tris, 100 mM NaCl, 0.1 mM TCEP, 5% glycerol, and pH 7.5). Five microliters of SIRT6, nucleosomes, or the SIRT6:nucleosome complex was diluted into 20 µL D_2_O in exchange buffer (50 mM HEPES, pH 7.4, 150 mM NaCl, 5 mM MgCl_2_, and 2 mM DTT) and incubated for various HDX time points (e.g., 0, 30, 60, 300, 600, 900, 1800, and 3600 s) at 4 °C and quenched by mixing with 25 µL of ice-cold 4 M guanidine hydrochloride, 1% trifluoroacetic acid. The sample tubes were immediately placed on dry ice after the quenching reactions until the samples were injected into the HDX platform. Upon injection, samples were passed through an immobilized pepsin column (2 mm × 2 cm) at 200 µL/min and the digested peptides were captured on a 2 mm ×  1 cm C8 trap column (Agilent) and desalted. Peptides were separated across a 2.1 mm × 5 cm C18 column (1.9 µm Hypersil Gold, ThermoFisher) with a linear gradient of 4–40% CH_3_CN and 0.3% formic acid over 5 min. Sample handling, protein digestion, and peptide separation were conducted at 4 °C. Mass spectrometric data were acquired using an Orbitrap mass spectrometer with a measured resolving power of 65,000 at *m*/*z* 400. Three biological replicates were repeated. HDX analyses were performed in duplicate or triplicate, with single preparations of each protein:ligand complex. The intensity weighted mean *m*/*z* centroid value of each peptide envelope was calculated and subsequently converted into a percentage of deuterium incorporation. Corrections for back-exchange were made on the basis of an estimated 70% deuterium recovery, and accounting for the known 80% deuterium content of the deuterium exchange buffer. When comparing the two samples, the perturbation %D is determined by calculating the difference between the two samples. HDX Workbench (v4.5) colors each peptide according to the smooth color gradient HDX perturbation key (%D) shown in each indicated figure. Differences in %D between −5 to 5% are considered non-significant and are colored gray according to the HDX perturbation key^60^. In addition to the −5 to 5% test, unpaired *t*-tests are calculated to detect statistically significant (*p* < 0.05) differences between samples at each time point. At least one time point with a *p* value < 0.05 was present in the data set further confirming that the difference is significant.

### Nuclear magnetic resonance

NMR spectra on SIRT6^(270–355)^ were recorded on a Bruker Avance III spectrometer operating at 900 MHz (^1^H) and equipped with a triple-resonance cryogenic probe. The temperature of the sample was regulated at 25 °C during the experiment. The one-dimensional proton spectrum was recorded using excitation sculpting for suppression of the signal from bulk water, with a repetition delay of 1.0 s, using a spectral window of 16 ppm in the ^1^H dimension and 16,384 complex points. The FID was accumulated 256 times and processed in TopSpin for inspection.

### Bioinformatics

Predictions of disorder along the SIRT6 sequence was performed using MetaPrDOS, which uses a meta-bioinformatics approach that comprises seven independent programs: PrDOS, DISOPRED2, DisEMBL, DISPROT(VSL2P), DISpro, IUpred, and POODLE-S^61^. Theoretical pIs were calculated by the ProtParam tool on ExPASy^62^, and percent identity between residues was calculated by ClustalW (v2.1)^63^.

### Differential scanning fluorometry

For each experiment, 10 µM of SIRT6 or SIRT6 mutants was equilibrated with 3.75x Sypro Orange on ice for 30 min in 20 mM potassium phosphate, pH 7.5. Fluorescence was monitored on a Bio-Rad CFX96 real-time thermocycler during a 0.5 °C/min gradient.

### HPLC deacetylation assay

To compare activity between mutants, 10 µM SIRT6 or SIRT6 mutants were incubated with 200 µM H3K9ac peptide (KQTARK(ac)STGGKAPRWW) in 20 mM potassium phosphate, pH 7.5 at 37 °C. Catalysis was initiated with 0.5 mM NAD^+^, then quenched with 2% TFA after 30 min. The product was separated using reversed phase-HPLC on a Kinetex C18 column (Phenomenex). The product and substrate was monitored at 214 nm during a HPLC gradient from 33 to 100% B (30% acetonitrile, 0.05% TFA) for 25 min at 1.5 mL/min. Each assay was repeated four times.

### Cell culture

The pCDNA3.1 plasmid expressing FLAG-tagged, wild-type SIRT6 was a generous gift from Raul Mostoslavsky^10^. HCT116 and 293T cells were grown in 1640 RPMI (Life Technologies) supplemented with 10% FBS (Life Technologies). To introduce SIRT6^Δ293–344^ and SIRT6^Δ302–344^ mutations into the vector, back-to-back primers were used to amplify the sequence N-terminal to residue 292 or 301 and C-terminal to 344, thereby removing the sequence in between:

SIRT6^Δ293–344^ Forward primer: CCCAAAAGGGTGAAGGCCAAGGCGG

SIRT6^Δ293–344^ Reverse primer: CGGGCGGGGCAGGGGTGG

SIRT6^Δ302–344^ Forward primer: CCCAAAAGGGTGAAGGCC

SIRT6^Δ302–344^ Reverse primer: CTCCTTGGGCTCCAGCTT

### Transfection and chromatin extraction

Empty vector or pCDNA3.1 plasmids expressing the FLAG-tagged SIRT6 or mutant proteins were transiently transfected in 293T cells with Lipofectamine 3000 (Thermo Fisher) according to the manufacturer’s instructions. After 48 h, the cells were lysed with lysis buffer (10 mM HEPES, 10 mM KCl, 0.05% NP-40, and pH 7.4) and protease inhibitors. The cells were then centrifuged at 18,000×*g* to pellet the nuclei from the soluble cytoplasm. The nuclei was then treated with low salt buffer (10 mM Tris, 0.2 mM MgCl_2_, 1% Triton X-100, and pH 7.4) and centrifuged again to separate the soluble nucleoplasm and insoluble chromatin. The chromatin fraction was resuspended in SDS/βME buffer for further immunoblot analysis. Gels were stained with Revert total protein stain (LI-COR), then blotted for H3K9ac (Active Motif 39917) and FLAG (Cell Signaling 2368). If possible, the membranes were stripped and re-blotted for total H3 (Abcam ab46765). All primary antibodies were used at 1:5000 dilutions. The secondary antibody used was anti-rabbit IRDye 800CW (1:7500 dilution; LI-COR #925–32211). A protein ladder (Precision Plus; Bio-Rad) was typically included in the gels. The fluorescent blots were visualized on an Odyssey Imager (model #9120) using Odyssey Software (v3.0.30). The bands were quantified in Image Studio Lite (v5.2.5; LI-COR): each H3K9ac signal was normalized to either total H3 signal or total histone signal (around 15–20 kDa) from Revert staining. The level of H3K9ac relative to the wild-type SIRT6 condition was reported. The data points represent three different transfection experiments. All uncropped blots are provided in the source data file.

### HCT116 chromatin preparation and activity assays

To prepare endogenous nucleosomes, 4 µM TSA was added to HCT116 cells 24 h before harvest to preserve protein acetylation. The cells were harvested by trypsinization and pelleted. In all, 6.7 × 10^6^ cells were resuspended in 1 mL digestion buffer (50 mM Tris, 1 mM CaCl_2_, 0.2% Triton X-100, and pH 7.6) supplemented with protease inhibitors. For each reaction, 75 units of MNase (Worthington) was added, and the reaction was allowed to proceed for 5 min at 37 °C. The reaction was stopped with 5 mM EDTA and sonicated on a Covaris ultrasonicator using two cycles of the following settings: Peak Power - 75, Duty Factor - 5, Cycles/Burst - 100, Duration - 30 s on, 3 s off. To assess digestion on an agarose gel, 150 µL of digested product was treated with Proteinase K for 2 h at 55 °C. To assess the impact of SIRT6 binding on MNase digestion, 10 µM of SIRT6 or SIRT6^(1–301)^ was added to the cell lysate for one hour prior to MNase treatment.

To examine H3K9ac deacetylation, 100 nM SIRT6 or SIRT6 mutants was incubated with 0.5 mM NAD^+^ and native nucleosomes (25 µg total DNA content) in 500 µL reactions on ice. For the LANA peptide competition experiment, 50 µM peptide (or vehicle) was added to the nucleososomes for 30 min on ice. The reactions were initiated with enzyme addition and quenched at each time point with SDS/βME buffer. Because SIRT6^(1–292)^ proceeded more slowly than wild-type enzyme on peptide subtrate (Supplementary Fig. 1c; 14 nmol/min), the reactions proceeded for 90 min to ensure ample time for deacetylation. The samples were resolved via 4–15% gradient SDS-PAGE, stained for total protein with Revert solution, then blotted for H3K9ac. The H3K9ac bands were normalized to either H3 signal from the anti-H3 blot, or the total histone signal (~15–20 kDa) from Revert staining. Each assay was repeated at least three times. All uncropped blots are provided in the source data file.

### Reporting summary

Further information on research design is available in the Nature Research Reporting Summary linked to this article.

## Supplementary information

Supplementary Information

Peer Review File

Reporting Summary

## Data Availability

The data supporting the findings of this study are available from the corresponding author upon reasonable request. HDX data are available in Figshare [10.6084/m9.figshare.12937103.v1]. Figures of nucleosomes and SIRT6 were generated from publicly available datasets from the Protein Data Bank (nucleosome: 3LZ0 [10.2210/pdb3lz0/pdb]; SIRT6: 3PKJ [10.2210/pdb3pkj/pdb]). Source data are provided with this paper.

## Source data

Source Data
