# Supplementary material for: Multivalent interactions drive nucleosome binding and efficient chromatin deacetylation by SIRT6
Source: Nat Commun. 2020 Oct 16;11:5244. doi: 10.1038/s41467-020-19018-y (PMC7568541; doi:10.1038/s41467-020-19018-y)
Supplement: Supplementary file 1 — Supplementary Information [file 41467_2020_19018_MOESM1_ESM.pdf]

Supplementary Information

Multivalent Interactions Drive Nucleosome Binding and Efficient Chromatin Deacetylation by SIRT6

Liu et al.

Supplementary Figure 1

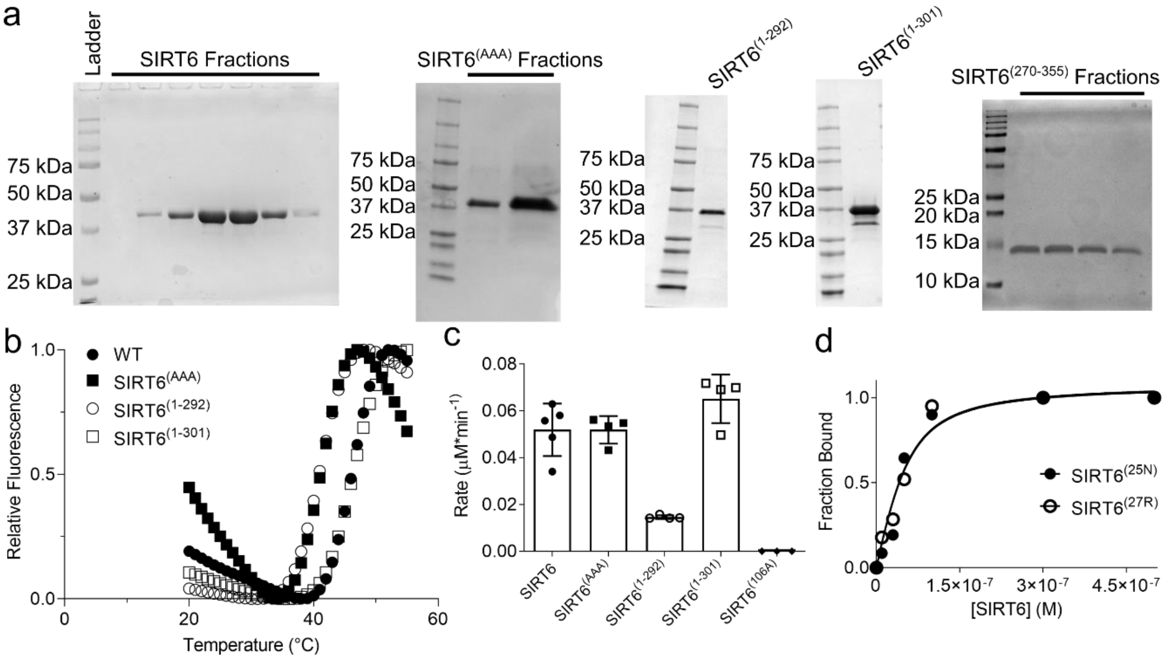

Proteins used in this study. a) Coomassie blue-stained SDS PAGE of recombinant SIRT6 and SIRT6 mutants. Each image is representative of three independent experiments. b) Differential scanning fluorometry (DSF) was used to ascertain that all mutant proteins did not have dramatically perturbed structure. c) To ensure that all proteins were catalytically active, proteins were allowed to deacetylate a peptide substrate bearing H3K9ac. Data are presented as mean ± s.d. from 4 independent experiments (3 for SIRT6$^{(106A)}$). d) SIRT6 mutations at the N-terminus (25N or 27R) did not alter NCP binding ability (n = 1; $K_{D(app)}$ for both mutants = 14 nM). Source data are provided as a source data file.

Supplementary Figure 2

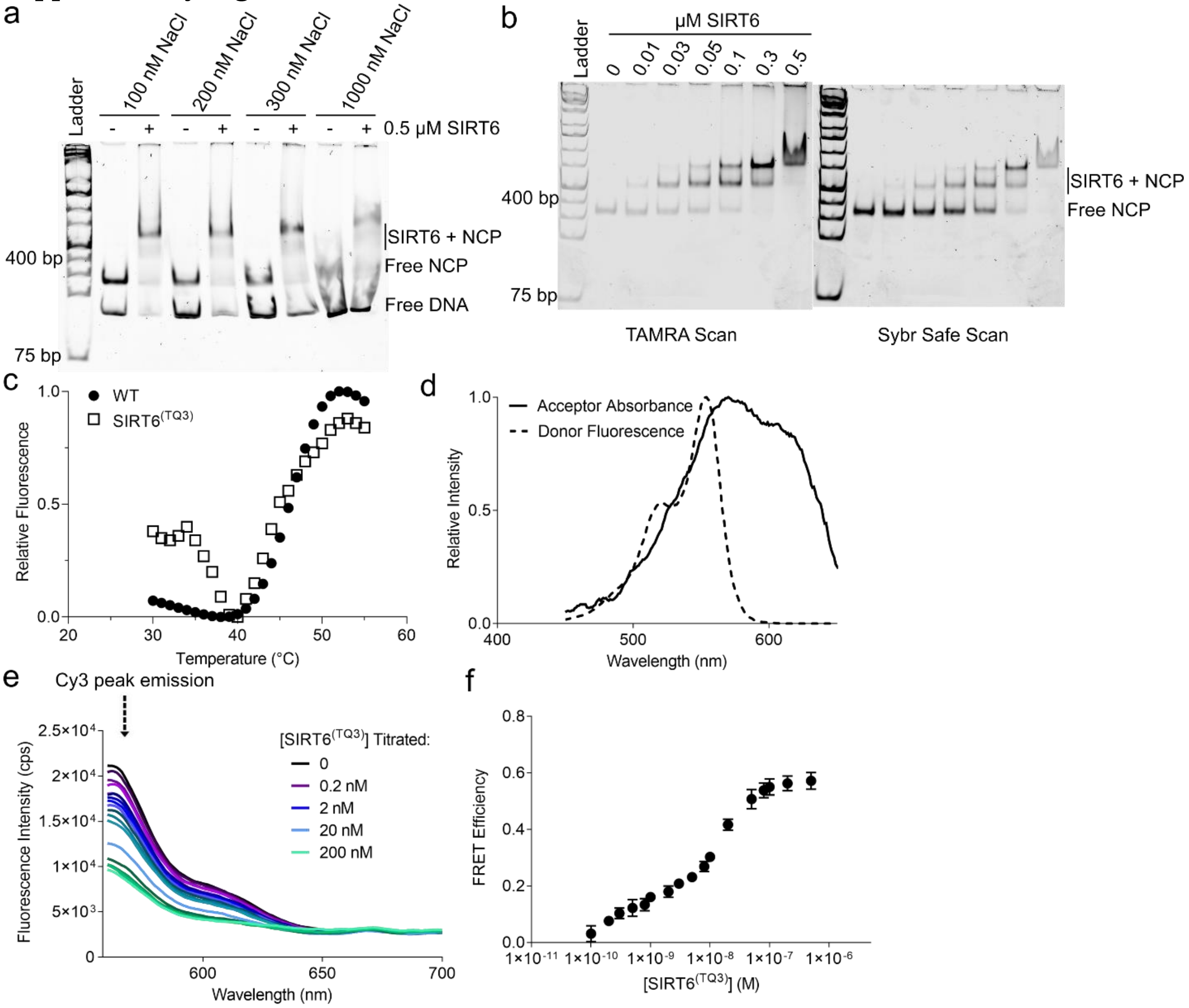

Native gel and FRET analysis of SIRT6:NCP interactions. a) SIRT6:NCP complexes were analyzed in various ionic strength conditions in EMSAs. The image is representative of two independent experiments. b) A fluorescent EMSA was developed to quantitatively determine the stoichiometry of SIRT6:NCP complexes. The gel was stained with SYBR Safe for DNA, then scanned for both TAMRA fluorescence from SIRT6$^{(TAMRA)}$ and SYBR Safe fluorescence. The images are representative of 3 independent experiments. c) DSF of SIRT6$^{(TQ3)}$ reveals that protein labeling does not affect protein stability. d) A FRET assay was employed to measure SIRT6:NCP binding. Spectral overlap of Cy3 (donor) fluorescence and TQ3 (acceptor) absorbance. e) Spectra of Cy3 fluorescence upon titration of SIRT6$^{(TQ3)}$ into Cy3-labeled nucleosomes. f) FRET efficiency was calculated from loss of donor emission. Data are presented as mean ± s.d. from 4 independent experiments. Source data are provided as a source data file.

Supplementary Figure 3

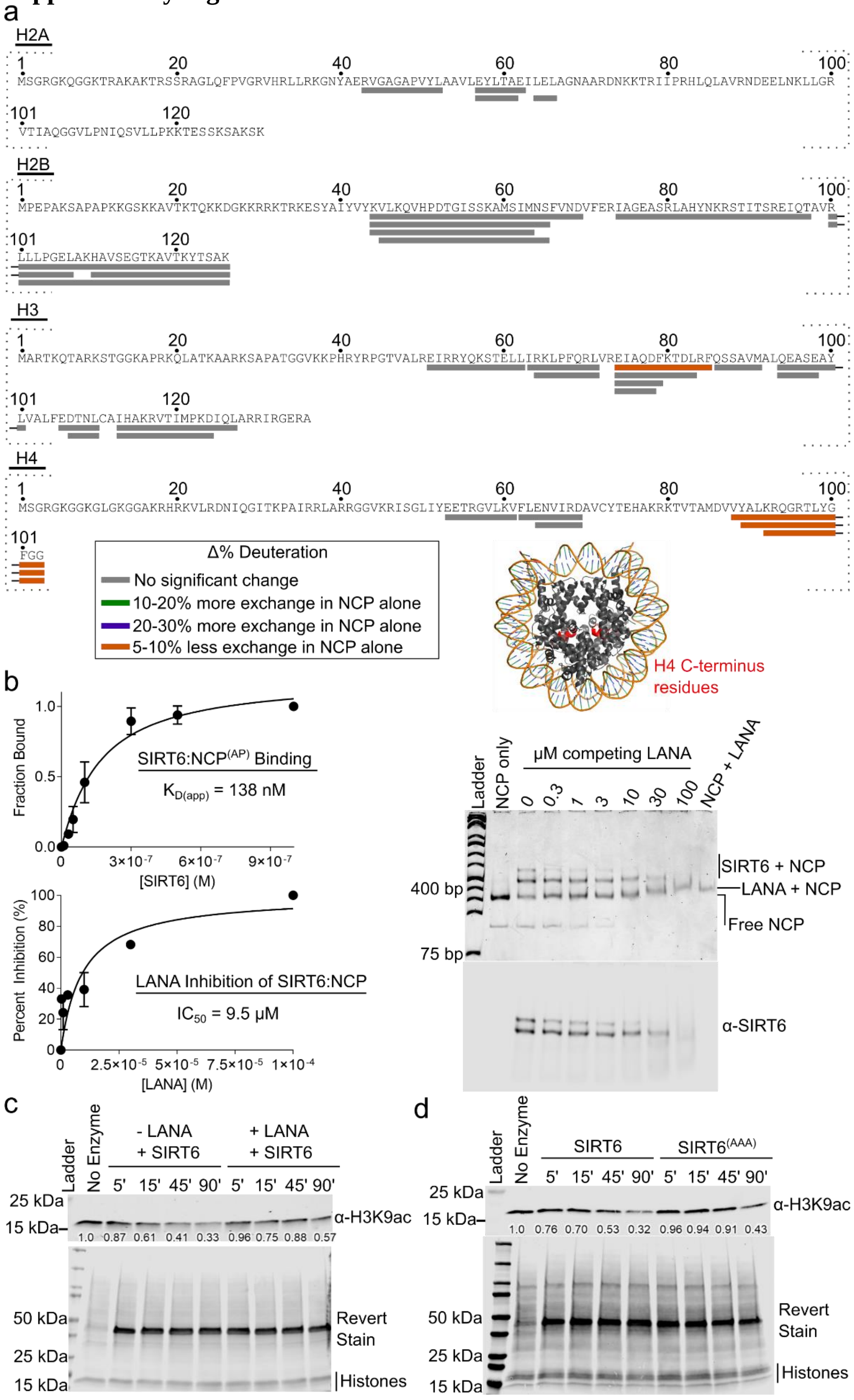

HDX analysis of histone peptides and confirmation of SIRT6:nucleosome binding sites. a) Peptides identified for H2A, H2B, H3, and H4 are colored according to changes in deuteration. Orange indicates that the peptide exchanged 5-10% less when it was in nucleosomes alone versus in a SIRT6:nucleosome complex. b) The $K_{D(app)}$ for the SIRT6:NCP$^{(AP)}$ interaction shows that the mutated NCP has weaker affinity for SIRT6. Data are presented as mean ± s.d. from 3 independent experiments. The LANA competition experiment (IC_{50} = 9.5 µM) (Fig. 2b) was immunoblotted for SIRT6 to confirm SIRT6 displacement. Data are presented as mean ± s.d. from 4 independent experiments. c) 50 µM LANA peptide or vehicle was added to HCT116-derived nucleosomes before SIRT6 addition. Deacetylation of H3K9ac was then monitored at the indicated time points by immunoblotting. d) Comparison of deacetylation activity between SIRT6 and SIRT6$^{(AAA)}$ on HCT116-derived nucleosomes. The numbers below each band indicate the H3K9ac level relative to the no enzyme control, normalized to histone signal detected by Revert stain (15-20 kDa). The images in c) and d) are each representative of 3 independent experiments. Source data are provided as a source data file.

Supplementary Figure 4

a

CTD	Length (aa)	% ID with SIRT6 CTD	% Prolines	Theoretical pI
SIRT1	249	25	7.6	4.3
SIRT2	50	34	14	5.7
SIRT6	83	-	25.3	10.4
SIRT7	69	30	8.7	10.6

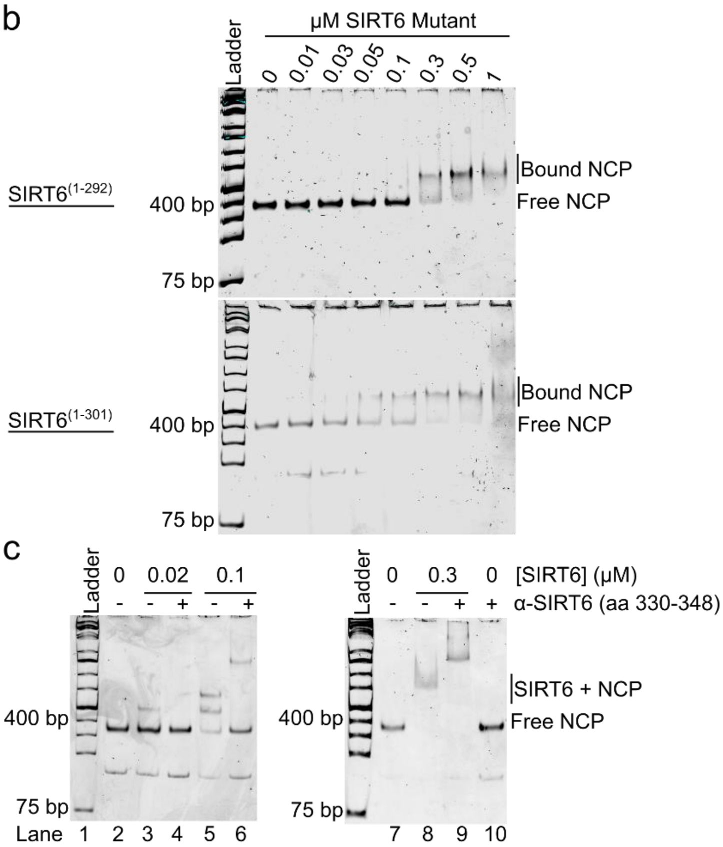

The SIRT6 CTD is unique among sirtuins and participates in nucleosome binding. a) Comparison of C-terminal domain sequences and known features among the four human nuclear sirtuins. Percent identity was determined by ClustalW and theoretical pI was calculated by the ProtParam tool on ExPASy. b) Native gel analysis of SIRT6$^{(1-292)}$ and SIRT6$^{(1-301)}$ binding to 50 nM NCPs. The images are each representative of 3 independent experiments. c) An antibody specific for SIRT6 residues 330-348 (Abcam 62738) was used to assess CTD binding to the high and low affinity sites. When the antibody is pre-incubated with 20 nM SIRT6, which only occupies the high affinity site, binding is lost (lanes 2 and 3). The antibody does not appear to disrupt formation of the 2:1 SIRT6:nucleosome complex, indicating that the CTD is only essential for part of a single binding event. The images are representative of 3 independent experiments.

Supplementary Figure 5

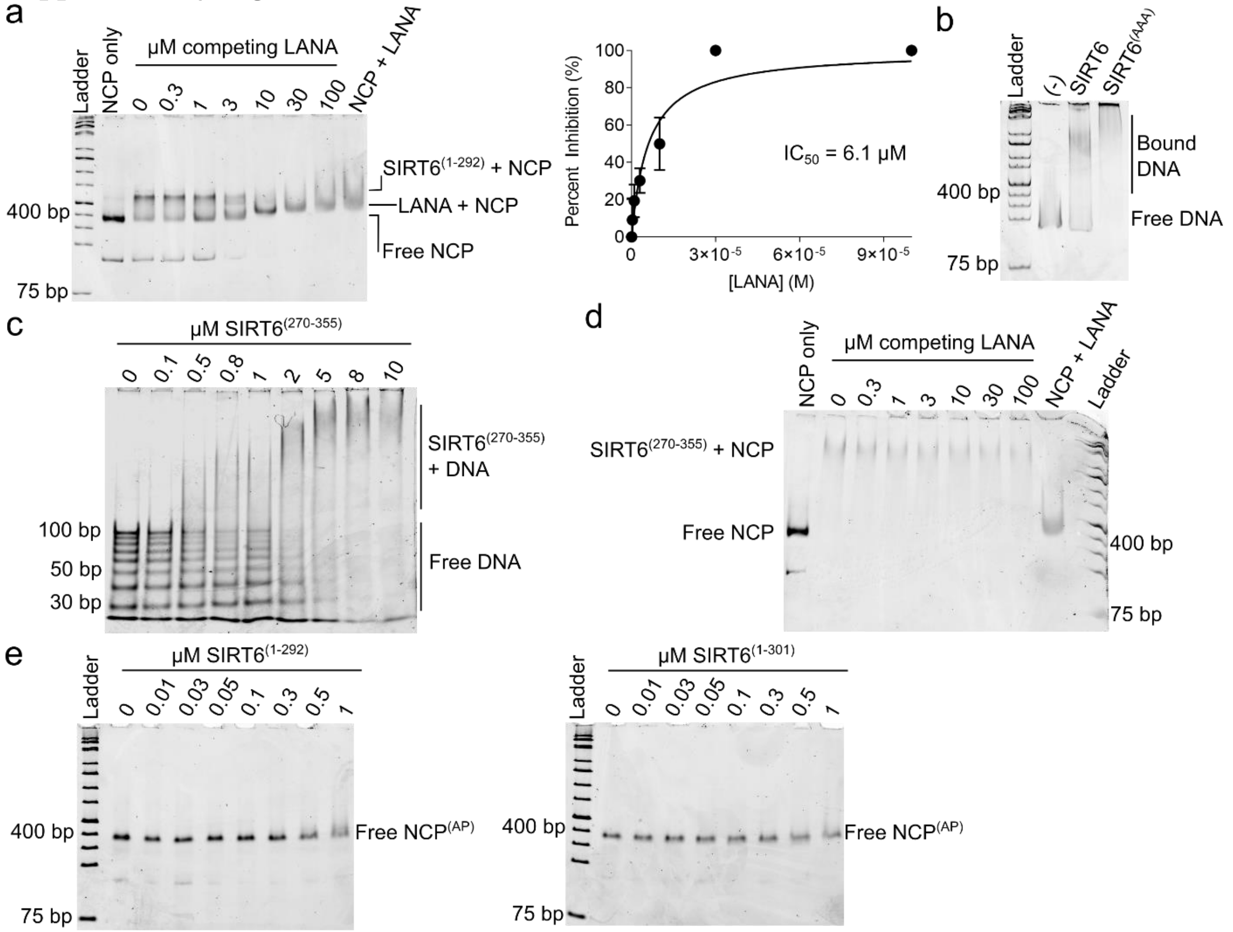

Analysis of CTD binding and function. a) LANA competition of the SIRT6$^{(1\text{-}292)}$:NCP complex has a similar IC_{50} value as observed with full-length SIRT6 (Supplementary Fig. 3b). Data are presented as mean ± s.d. from 3 independent experiments. b) EMSA of 50 nM 601 sequence DNA interacting with 500 nM SIRT6 or SIRT6$^{(AAA)}$. The mutant SIRT6 does not exhibit impaired DNA binding. The image is representative of 3 independent experiments. c) EMSA of the CTD binding to a 10 step DNA ladder demonstrates that the CTD can accommodate various DNA lengths and sequences. The image is representative of 3 independent experiments. d) EMSA of 500 nM SIRT6$^{(270\text{-}355)}$ bound to 50 nM NCP, then incubated with the indicated LANA concentrations. LANA does not compete CTD interactions with nucleososomes, which reveals that the CTD is not involved with acidic patch binding. The image is representative of 3 independent experiments. e) EMSAs of C-terminally truncated SIRT6 mutants binding to NCP$^{(AP)}$ reveals that no specific complexes are formed when both acidic patch-dependent and CTD-dependent interactions are unavailable. Each image is representative of 3 independent experiments. Source data are provided as a source data file.

Supplementary Figure 6

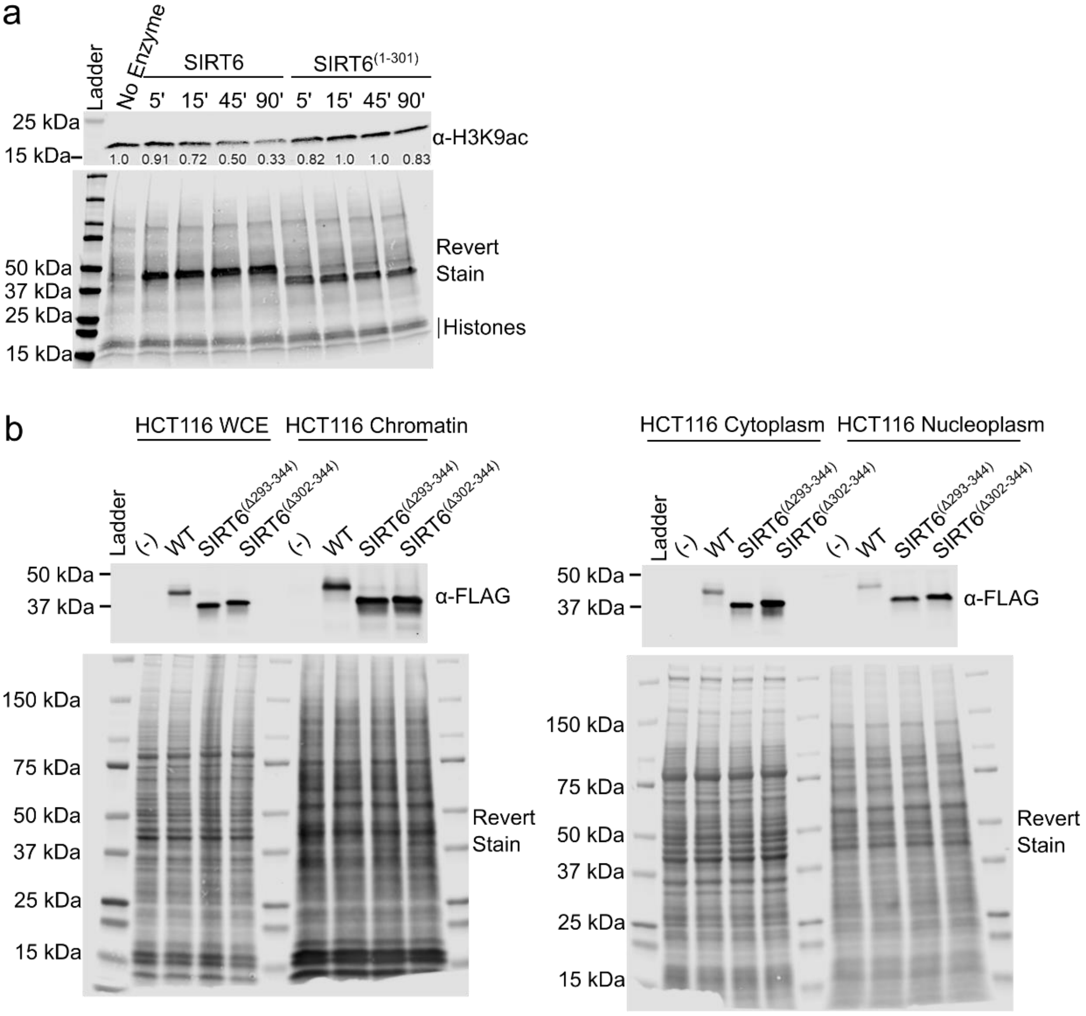

Activity and cellular localization of C-terminally truncated SIRT6. a) Immunoblot analysis of SIRT6 and SIRT6$^{(1\text{-}301)}$ activity on HCT116-derived nucleosomes. 100 nM of SIRT6 or SIRT6$^{(1\text{-}301)}$ was incubated with NAD^+ and nucleosomes, quenched at various time points, and assessed for H3K9ac levels by immunoblotting. The numbers below the bands indicate the H3K9ac signal relative to the no enzyme control, all normalized to Revert-stained histones (15-20 kDa). b) The sub-cellular localization of SIRT6 and SIRT6 mutants with C-terminal deletions was evaluated by biochemical fractionation of HCT116 cells. Cells were transfected with empty vector, wild-type SIRT6, or the indicated mutants. The nuclear localization signal (residues 345-355) was retained in the mutants, which displayed greater relative localization in the cytoplasm and nucleoplasm fractions. Source data are provided as a source data file.
