# Supplementary material for: Multivalent interactions drive nucleosome binding and efficient chromatin deacetylation by SIRT6
Source: Nat Commun. 2020 Oct 16;11:5244. doi: 10.1038/s41467-020-19018-y (PMC7568541; doi:10.1038/s41467-020-19018-y)
Supplement: Supplementary file 3 — Reporting Summary [file 41467_2020_19018_MOESM3_ESM.pdf]

nature research

Corresponding author(s): John Denu
Last updated by author(s): Sep 14, 2020

Reporting Summary

Nature Research wishes to improve the reproducibility of the work that we publish. This form provides structure for consistency and transparency in reporting. For further information on Nature Research policies, see Authors & Referees and the Editorial Policy Checklist .

Statistics

For all statistical analyses, confirm that the following items are present in the figure legend, table legend, main text, or Methods section.

n/a	Confirmed	
☐	☒	The exact sample size (*n*) for each experimental group/condition, given as a discrete number and unit of measurement
☐	☒	A statement on whether measurements were taken from distinct samples or whether the same sample was measured repeatedly
☒	☐	The statistical test(s) used AND whether they are one- or two-sided<br>*Only common tests should be described solely by name; describe more complex techniques in the Methods section.*
☒	☐	A description of all covariates tested
☒	☐	A description of any assumptions or corrections, such as tests of normality and adjustment for multiple comparisons
☐	☒	A full description of the statistical parameters including central tendency (e.g. means) or other basic estimates (e.g. regression coefficient) AND variation (e.g. standard deviation) or associated estimates of uncertainty (e.g. confidence intervals)
☒	☐	For null hypothesis testing, the test statistic (e.g. *F*, *t*, *r*) with confidence intervals, effect sizes, degrees of freedom and *P* value noted<br>*Give P values as exact values whenever suitable.*
☒	☐	For Bayesian analysis, information on the choice of priors and Markov chain Monte Carlo settings
☒	☐	For hierarchical and complex designs, identification of the appropriate level for tests and full reporting of outcomes
☒	☐	Estimates of effect sizes (e.g. Cohen's *d*, Pearson's *r*), indicating how they were calculated

Our web collection on statistics for biologists contains articles on many of the points above.

Software and code

Policy information about availability of computer code

Data collection	Typhoon FLA 9000 Control Software (v1.1), Odyssey Software (v3.0.30), FelixGX (v4.0)
Data analysis	Prism (v8), PyMOL (v2.3.0), Image J (v1.52d), Mascot (v2.3.01), HDX Workbench (v4.5), Image Studio Lite (v5.2.5), MetaPrDOS, ProtParam, ClustalW (v2.1)

For manuscripts utilizing custom algorithms or software that are central to the research but not yet described in published literature, software must be made available to editors/reviewers. We strongly encourage code deposition in a community repository (e.g. GitHub). See the Nature Research guidelines for submitting code & software for further information.

Data

Policy information about availability of data

All manuscripts must include a data availability statement. This statement should provide the following information, where applicable:
- Accession codes, unique identifiers, or web links for publicly available datasets
- A list of figures that have associated raw data
- A description of any restrictions on data availability

The data supporting the findings of this study are available from the authors upon reasonable request. Source data are provided with this paper for Fig. 1a, b, c, d, 2e, 3a, c, d, 4c, 5b, c, and Supplementary Fig. 1b, c, d, 2c, d, e, 3b, c, d, 5a, 6a, b. HDX data are available in Figshare [https://doi.org/10.6084/m9.figshare.12937103.v1]. Figures of nucleosomes and SIRT6 were made from publicly available datasets from the Protein Data Bank (nucleosome: [https://www.rcsb.org/structure/3lz0]; SIRT6: [https://www.rcsb.org/structure/3pkj]).

Field-specific reporting

Please select the one below that is the best fit for your research. If you are not sure, read the appropriate sections before making your selection.

☒ Life sciences ☐ Behavioural & social sciences ☐ Ecological, evolutionary & environmental sciences

For a reference copy of the document with all sections, see nature.com/documents/nr-reporting-summary-flat.pdf

Life sciences study design

All studies must disclose on these points even when the disclosure is negative.

Sample size	In order to report mean and standard deviation, sample size was n = 3 or greater. This criteria was sufficient to determine binding constants.
Data exclusions	No data were excluded from analyses.
Replication	All experiments were independently conducted at least three times and successfully reproduced, often with different batches of protein.
Randomization	Randomization was not used because no clinical trials or population studies were conducted in this study.
Blinding	Blinding was not applicable as no clinical trials or population studies were conducted in this study.

Reporting for specific materials, systems and methods

We require information from authors about some types of materials, experimental systems and methods used in many studies. Here, indicate whether each material, system or method listed is relevant to your study. If you are not sure if a list item applies to your research, read the appropriate section before selecting a response.

Materials & experimental systems

n/a	Involved in the study	
☐	☒	Antibodies
☐	☒	Eukaryotic cell lines
☒	☐	Palaeontology
☒	☐	Animals and other organisms
☒	☐	Human research participants
☒	☐	Clinical data

Methods

n/a	Involved in the study	
☒	☐	ChIP-seq
☒	☐	Flow cytometry
☒	☐	MRI-based neuroimaging

Antibodies

Antibodies used	anti-SIRT6: Abcam #ab62739 (Lot GR3211017-1). Immunoblot: 1:5000. Binding competition: 0.04 mg/mL<br>anti-SIRT6: Abcam #ab62738 (Lot GR8248-7). Binding competition: 0.15 mg/mL<br>anti-FLAG: Cell Signaling #2368 (Lot 12 Ref. 01/2019). Immunoblot: 1:5000<br>anti-H3K9ac: Active Motif #39917 (Lot 06419005). Immunoblot: 1:5000<br>anti-H3: Abcam #ab46765 (Lot GR13524-1). Immunoblot: 1:5000<br>anti-rabbit IRDye 800CW: LI-COR #925-32211. Immunoblot: 1:7500
Validation	All antibodies were validated by the respective manufacturers. Abcam #ab62739 was validated through ChIP and competition with immunizing peptide. Abcam #ab62738 was validated through competition with immunizing peptide. Active Motif #39917 was validated through ChIP-seq. Abcam #ab46765 was validated through ChIP. Cell Signaling #2368 was validated through transfections (with negative and positive controls) followed by immunoblotting.

Eukaryotic cell lines

Policy information about cell lines

Cell line source(s)	293T from ATCC<br>HCT116 from ATCC
Authentication	The cell lines were authenticated by ATCC, which authenticates each cell line with "morphology, karyotyping, and PCR based approaches to confirm the identity of human cell lines and to rule out both intra- and interspecies contamination (https://www.atcc.org/CellAuthenticationMatters.aspx)." The cell lines were not authenticated in the authors' laboratory.

Mycoplasma contamination	All cell lines tested negative for mycoplasma.
Commonly misidentified lines<br>(See ICLAC register)	No commonly misidentified cell lines were used in this study.
