# Supplementary material for: Multivalent interactions drive nucleosome binding and efficient chromatin deacetylation by SIRT6
Source: Nat Commun. 2020 Oct 16;11:5244. doi: 10.1038/s41467-020-19018-y (PMC7568541; doi:10.1038/s41467-020-19018-y)
Supplement: Supplementary file 2 — Peer Review File [file 41467_2020_19018_MOESM2_ESM.pdf]

Reviewers' comments:

Reviewer #1 (Remarks to the Author):

The manuscript by Liu et al describes a biophysical and biochemical description of the interaction between SirT6 and nucleosomes. While it certainly provides details of interest to the specialists in this field, I feel that progress in the field and relevance to a wider readership are limited and it might be better suited for a more specialized journal.

The study leaves many questions unanswered. Is the high affinity relevant in cells? As discussed by the authors, the affinity might be modulated, for example by protein modifications. So it would be important to know whether this very high affinity is normally observed in a cellular environment? A first step would be localization - Sirt6 should be nowhere else than on nucleosomes. And is the specificity for nucleosomes - if observed - due to the second, weaker binding site? Or does SirT6 also colocalize with DNA transcripts? What about RNA? And does the SirT6-C-terminus releave nucleosome binding - the authors have the peptide and could test that at least in vitro. But my main concern is that there is no experimental work here showing that all this applies to the cellular system.

A second concern is that the model of the authors remains very "fluffy". What causes the second CTD to be accessible rather than to engage in the high affinity interaction?! Based on their mapping of interaction sites on both binding partners, what would the complex look like - does it provide a potential answer? Is the 2:1 complex related to the one recently described in the Elife paper by the Toiber group?

The authors postulate that the histone H4 core is destabilized upon complex formation based on HDX data. How can I imagine such a change in the compact structure of the nucleosome? Has anything like that been described before? And even more important: What are the basal levels of HDX over the sequences of the two partners and what are the errors of determined exhange rates? How many times were they reproduced? Presentation of the HDX data definitely needs to include more detail.

Smaller points:
line 173 - claim that mutated residues contribute to nucleosome binding; it would be good to mention that the contribution can be indirect.
line 181 - an antibody is very, very bulky, can shield other regions and disrupt folding. The result thus doesn't really show involvement of the N-terminal helix in binding. More conclusive would maybe be a competition experiment with N-terminus as free peptide.

Figure 2e - cannot see any effect, only in the numbers?! And even there, at later times differences are minimal - how often was this reproduced?!
Is the SirT6 C-terminus also unstructured in the full-length context? Are there, for example, CD data for full-length and C-terminal truncated protein?

Reviewer #2 (Remarks to the Author):

In this manuscript, Liu et al., proposed the mechanism by which human SIRT6 interacts with the nucleosome. SIRT6 is a NAD+-dependent histone H3 lys9 deacetylase involved in longevity. Using biophysical and biochemical tools, such as EMSA, FRET, HDX, and mutational studies, authors claim that SIRT6 binds to the nucleosome via two asymmetric binding sites with 2:1 stoichiometry and showed that high affinity binding is driven by the association of the C-terminal disordered region of SIRT6 with the nucleosomal DNA. Although the authors proposed an interesting model by which Sirt6 recognizes the nucleosome, experimental evidence they provided was either indirect or insufficient to support their proposed mechanism without ambiguity. Therefore, I believe the authors must present more direct and solid evidence to support their model to publish their work.

Major issues

1. The main interaction between Sirt6 and the nucleosome seems to be driven by the C-terminal disordered region (CTD) of Sirt6, which contains several positively charged residues. The authors also mentioned that this Sirt6 CTD and the nucleosomal DNA interactions are the sequence non-specific. This is further supported by Suppl. Fig. 2a and 4b, where free DNA also interacts with Sirt6 and disappears in EMSA. Altogether, evidence indicates that Sirt6 CTD interacts strongly (several nM affinity level) to the nucleosomal DNA in a non-specific manner. This raises the concern that any interaction experiment performed here was mainly driven by CTD-DNA interactions, which might be independent to Sirt6 and nucleosomes. More specifically, Sirt6 CTD can interact with the nucleosome in a random manner via the nucleosomal DNA. Authors state that "We propose that the promiscuous DNA-binding function of the CTD, which can accommodate multiple forms of DNA, allows the protein to rapidly find nucleosomes within nuclear confines." on page 16-17. But, this also means that Sirt6 can bind any part of the genomic DNA, not restricted by the nucleosome. The authors also suggested that CTD may recruit other factors for its specific targeting. If Sirt6 CTD participates in nucleosome-specific recognition via other binding components, then this study should be assessed again in the presence of these factors in order to be biologically meaningful.
2. Authors performed the HDX experiment to identify the interface between Sirt6 and the nucleosome. I think this is a valuable approach; however, the HDX result does not match well with the current model that the authors proposed. For example, the authors proposed that the NBR region of Sirt6 interacts with the acidic patch region of the nucleosome. However, histones H2A and H2B at the acidic patch region did not show the protection upon Sirt6 interaction. The key supporting data is Sir6AAA (triple mutations of S45A/N206A/D208A), which shows seven fold decrease in binding (however, still fairly tight binding with Kd=97nM). I believe seven fold decrease with triple point mutations indicates a mild effect. Probably, affinity measurement using CTD-deleted Sirt6 1-292 with this triple mutation (AAA) may provide a more realistic affinity change between NBR and the nucleosome (without considering the effect of DNA-nucleosome interaction by CTD). In parallel, mutations in acidic patch residues to monitor the affinity change would be necessary. The LANA peptide competition assay is, in my opinion, indirect evidence and LANA binding can sterically restrict the binding of Sirt6 interaction broadly toward the nucleosome. I don't think this can support the direct interaction of Sirt6 to the acidic patch region of the nucleosome.
3. Another key interacting region of Sirt6, CTD, did not show the result in the HDX experiment. Two major contacting regions, CTD and nucleosomal DNA as well as NBR and the acidic patch, are only partially demonstrated, which allows the other interpretations, such as NBR may binds other histone regions other than the acidic patch
4. Cys18 of Sirt6 is positioned in the proximity to the helix (residues 66-73) and labeling with Tide Quencher 3(TQ3), a bulky compound, would most likely break this loop and helix interaction. If this is the case, the N-terminal loop region will be flexible /mobile and the FRET data interpretation must be re-evaluated and re-considered. I'd like to hear author's opinion about this.
5. Although 2:1 stoichiometry of Sirt6:nucleosome seems a valid claim, how do authors envision (or distinguish) this 2:1 stoichiometry as either two asymmetric bindings on one surface of the nucleosome or Sirt6 binds on both sides of the nucleosome (as shown in cryoEM structure of Dot1L-nucleosome)? I think it is possible that one Sirt6 binds to one side of the nucleosome using CTD-nucleosomal DNA for enhancing affinity and NBR-histones for specificity (two binding sites on one nucleosome surface). Asymmetric binding was proposed based on the FRET result. But the same interpretation is possible in case with each Sirt6 binds to each side of the nucleosome (only one end of nucleosomal DNA is labeled with Cy3 and the distance of Sirt6 Cys18 in both sides of the nucleosome will display different distance). If two Sirt6 molecules bind to one side of the nucleosome, why they don't bind the other side of the nucleosome?
6. The HDX signal change in H4 C-terminal region might be important to Sirt6 recognition and function. However, authors didn't examine any follow-up experiments. Is there any reason why?

Reviewer #3 (Remarks to the Author):

SIRT6 is an essential protein involved in numerous diverse pathways suggesting that it plays a general role in gene regulation. One way that SIRT6 exerts its function is through the deacetylation of histone tails. Histone tail post-translational modifications are an important way that eukaryotes regulate transcription of genes, and a better understanding of the biological consequences of histone modifications (and their removal) are desirable, making the subject matter of general interest to biologists.
The authors present very nice in vitro characterization of SIRT6. Experiments and interpretation are generally very good. However one downside is that the study falls short of adding significant biological insight into SIRT6 function. The authors probe the binding details for how SIRT6 binds a nucleosome core particle in vitro, demonstrating that two SIRT6 molecules interact with one nucleosome core particle through a two-stage process, and probe SIRT6 protein for the residues that are important for each binding stage. However, it is unclear how these observations translate to in vivo regulation. The authors state in the abstract that: "Unique among histone deacetylases, SIRT6 possesses the capacity to tightly interact with nucleosomes for efficient activity. The implications for SIRT6 targeting and regulation are discussed" yet none of the observations in this manuscript provide any insight into the specificity of interaction, and it is unclear how the enzyme would discriminate appropriate substrate from random nucleosomes in the nucleus.

We would like to thank all reviewers for the time and effort that they put into their insightful comments and useful critiques. In this response, we have made comments easy to identify by showing the reviewers' comments in bold italics, and our own responses in standard font.

REVIEWER 1

1) The manuscript by Liu et al describes a biophysical and biochemical description of the interaction between SirT6 and nucleosomes. While it certainly provides details of interest to the specialists in this field, I feel that progress in the field and relevance to a wider readership are limited and it might be better suited for a more specialized journal.

While we appreciate the reviewer's recognition that the mechanistic details are interesting, we have a philosophical difference regarding the idea that fundamental biochemistry belongs in specialist journals. We respectfully disagree that our results are for specialists. While there are numerous studies showing an important role of SIRT6 in longevity, cancer, and metabolism, there is comparably little understanding of the physical or biochemical mechanisms that drive these processes. Every approach has limitations, as every scientific approach has value. Without quantitative descriptions of SIRT6 interactions, the field is limited by the ambiguity inherent in over-expression and knockdown conditions, and inferring function. For this reason, we took a unique approach by focusing on rigorous biochemistry first, and then confirming the observations in select cellular experiments. Perhaps most critical for justifying a detailed biochemical/biophysical study: Prior results suggest that SIRT6 is tightly associated with chromatin, but no one knew how.

From our work here, we now understand the biochemical underpinnings of a unique nucleosome-binding mechanism, and the functionality of a previously uncharacterized SIRT6 domain. For both a basic understanding of SIRT6 biology and developing therapeutic strategies for modulating enzyme activity, researchers will need a detailed understanding of how the full-length enzyme interacts with nucleosomes. Our results explain how a single polypeptide chain can perform chromatin deacetylation at the global level.

2) The study leaves many questions unanswered. Is the high affinity relevant in cells? As discussed by the authors, the affinity might be modulated, for example by protein modifications. So it would be important to know whether this very high affinity is normally observed in a cellular environment? A first step would be localization – Sirt6 should be nowhere else than on nucleosomes... But my main concern is that there is no experimental work here showing that all this applies to the cellular system.

We would like to emphasize that many of these questions have already been addressed by other studies, which emphasized the importance of performing a series of biochemical and mechanistic investigations as we describe here. These studies include reports suggesting SIRT6 is associated with chromatin and is an efficient H3K9ac deacetylase in cells[1–3]. Therefore, our focus was to provide readers with a sound biochemical understanding of the intrinsic mechanisms that drive SIRT6 function. Along this line, our work demonstrates that SIRT6 alone harbors the capacity to interact with nucleosomes with high affinity and that this tight interaction endows the enzyme

with efficient deacetylation of nucleosomes. We provide compelling data regarding which parts of the nucleosome and SIRT6 are important for this interaction.

To be more explicit about these points, we have edited the manuscript to be clearer about how our in vitro experiments related to past biological work, and also how we can apply our findings to cellular function. Towards this end, we had previously showed that cells transfected with SIRT6 C-terminal mutants had weakened H3K9ac deacetylation activity, compared to wild-type transfected cells (Fig. 5c). In this revised manuscript, we have added new data, which show that transfected SIRT6 mutants with C-terminal deletions (but with retained NLS) exhibit comparably weaker localization to chromatin than wild-type SIRT6 (Supplementary Fig. 6b). Together, our study expounds on why SIRT6 is so enriched on chromatin and why SIRT6 is capable of reducing global levels of H3K9ac. The figure is reproduced below:

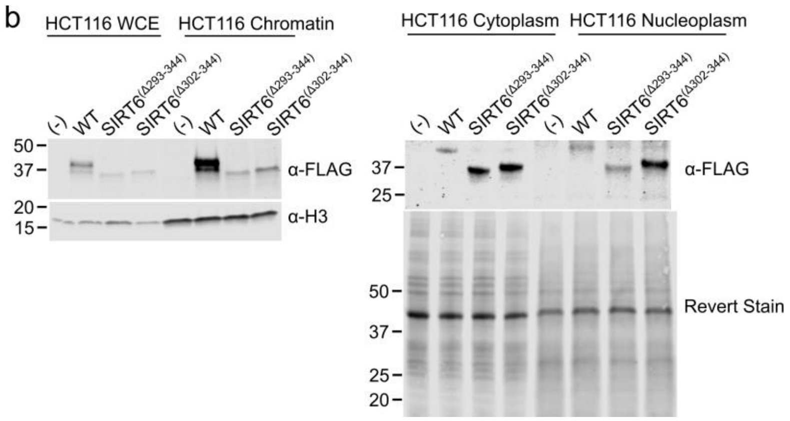

3) And is the specificity for nucleosomes – if observed – due to the second, weaker binding site? Or does SirT6 also colocalize with DNA transcripts? What about RNA?

The K_D of SIRT6 for nucleosomes is in the low nanomolar range, while DNA-binding through the CTD is only ~400 nM (Fig. 1a, 4c). This difference is due to the multivalent interactions available for nucleosome binding, which is the major conclusion of our study. Onn, et al. report no detectable binding to RNA[4]. We and others have shown that SIRT6 is primarily associated with chromatin; this is in accord with SIRT6 having high specificity for nucleosomes and not free RNA[1,2,5] (Supplementary Fig. 6b).

4) And does the SirT6-C-terminus releave nucleosome binding – the authors have the peptide and could test that at least in vitro. What causes the second CTD to be accessible rather than to engage in the high affinity interaction?!

We believe that the question of asymmetric binding, in which only one CTD is bound to the NCP, is a logical one that is consistent with a couple key points. The nucleosome structure itself exhibits asymmetric characteristics. In particular, the 601 sequence is non-palindromic, which promotes differences in local DNA flexibility, and causes asymmetric DNA unwrapping during disassembly[6,7]. Even in structural studies with palindromic DNA, the DNA gyres are asymmetric, and multiple nucleosomal DNA conformations exist in solution[8,9]. Furthermore, the NCP possesses 7 superhelical locations and a dyad, which have differing curvatures[10,11]. Solved

structures of protein:NCP interactions often show that DNA-binding domains have a preference for one site[10]. Consistent with this, the CTD in the context of full-length SIRT6 binds to a single site.

We also note that although the isolated CTD (residues 270-355) is an unrestricted DNA-binding domain, the CTD as part of the much larger SIRT6 protein is much more restrained. We reason that this steric restriction would also prevent the unbound CTD from engaging in nonspecific binding behavior. We believe that all these factors came into play when we observed the asymmetric binding. To expound on this discussion, we have added the following paragraph to the Discussion (Lines 371-377):

"Interestingly, only a single SIRT6 molecule employs the CTD for binding a nucleosome core particle, suggesting that the CTD is specific for a single nucleosomal DNA region. This mechanism would be consistent with the many nucleosome-binding proteins that have preference for specific regions on nucleosomal DNA, which is inherently asymmetric and features gyres of varying curvatures[8–11]. Although the isolated CTD exhibits promiscuous DNA-binding ability, the specific binding displayed when engaging NCPs suggests that the CTD is sterically restricted as part of the full-length SIRT6 protein, preventing non-specific DNA binding."

The CTD (residues 270-355) competition of SIRT6:NCP in EMSAs is a good recommendation to gain insight into the complex. We were able to test this, and found that addition of the CTD actually shifted SIRT6:NCP into nonspecific complexes. This result is consistent with the CTD being a promiscuous DNA binding domain by itself, but indicates that when the CTD is part of native SIRT6, the CTD is positioned to only engage one DNA region. Because of the multivalent interaction involving the NBR and the CTD of SIRT6, the CTD is sterically restricted. The data is shown below:

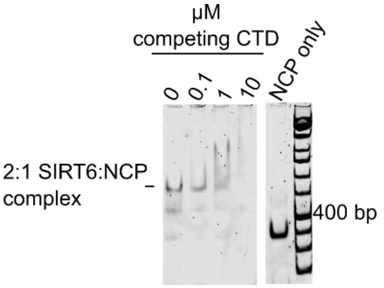

5) Based on their mapping of interaction sites on both binding partners, what would the complex look like – does it provide a potential answer?

In retrospect, we agree that our model figure needed to be clearer. The revised figure (Fig. 6 below) now shows both SIRT6 binding partners and their likely orientation given the collective data. Our data best supports a complex in which the N-terminal helix of the NBR participates in both interactions, and with the CTD interacting with nucleosomal DNA on only one face of the nucleosome.

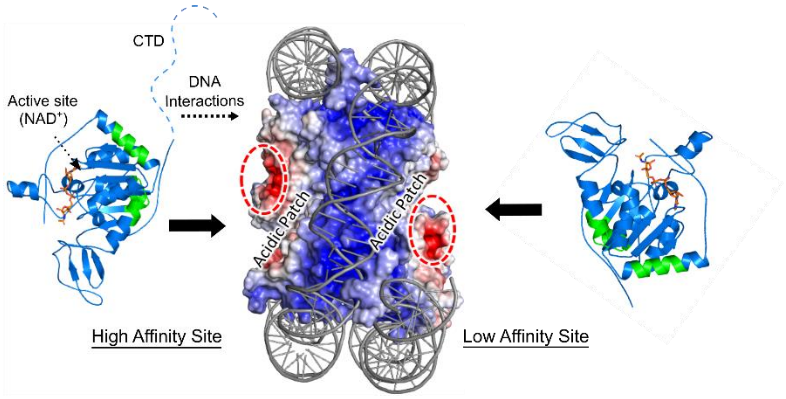

6) Is the 2:1 complex related to the one recently described in the Elife paper by the Toiber group?

We do not believe the 2:1 SIRT6:NCP complex described in our study is similar to the one proposed in the eLife paper, as the latter describes a ~45 base, single-stranded DNA complex with SIRT6[4]. Furthermore, the DNA binding site described by Onn, et al. is in the core of SIRT6. We do not observe any significant binding to DNA without the C-terminus under our conditions (Fig. 4a). The mutagenesis follow-up in the Onn, et al. paper do not show substantially weakened ssDNA binding. Overall, the experimental details, such as protein concentration used and the stability of the mutants, are not clear in the Onn, et al paper, making it difficult to interpret those results. Importantly, our study provides a detailed understanding of how SIRT6 binds the nucleosome.

7) The authors postulate that the histone H4 core is destabilized upon complex formation based on HDX data. How can I imagine such a change in the compact structure of the nucleosome? Has anything like that been described before?

Yes, the perturbance at the H4 C-terminus is surprising, but after thorough literature investigations, we note that recent studies show the octamer core is structurally plastic, and increased solvent exposure can be induced by HP1 and SNF2H[12,13]. This point is now addressed in Results Lines 142-148 and Discussion Lines 413-425:

Lines 142-148: “Nucleosomes that were bound to SIRT6 experienced more exchange in the H4 C-terminus (residues 87-102), indicating a possible structural destabilization induced by SIRT6 (Supplementary Fig. 3a). The H4 C-terminus is known to be structurally plastic, as recent studies of SNF2H- and HP1-bound NCPs revealed that this octamer region can experience increased solvent exposure due to protein binding[12,13]. Importantly, the H4 residues are buried in the canonical nucleosome structure, which suggests that SIRT6 interactions with other histone domains or nucleosomal DNA perturb the NCP structure.”

Lines 413-425: “Aside from the acidic patch, we also observe destabilization (increased exchange) at the H4 C-terminus (Supplementary Fig. 3a). This H4 domain is noted to have structural plasticity: it adopts a parallel β-sheet to interact with nucleosomal H2A, yet rotates almost 180° to form an anti-parallel sheet with the histone chaperone Asf1[14,15]. Partial deletion of

the H4 C-terminus leads to a destabilized nucleosome structure, while modifications near this domain lead to increased nucleosome unwrapping, consistent with a site that undergoes rapid DNA "breathing"[10,16,17]. Because the H4 C-terminal residues are buried in the nucleosome, the simplest explanation for SIRT6-induced destabilization is that SIRT6 interactions with histones and/or DNA perturb the nucleosome structure. In support, recent studies show that other proteins, including HP1 and the acidic patch-binding SNF2H, also increase octamer plasticity at the H4 C-terminus, revealing that even buried histone residues are susceptible to conformational changes as a result of protein-nucleosome binding[12,13]. Future work is needed to determine if the SIRT6-induced changes have functional consequences on chromatin structure."

8) And even more important: What are the basal levels of HDX over the sequences of the two partners and what are the errors of determined exhange rates? How many times were they reproduced? Presentation of the HDX data definitely needs to include more detail.

The HDX levels were measured with three biological replicates. Percent deuteration represented the number of measured deuterons incorporated by the peptide as a percentage of the total exchangeable hydrogen atoms for the peptide[18]. To calculate change in deuteration, we subtracted the percent deuteration of the two samples (SIRT6 and the complex) from each other. This presentation is a standard one for most journals and is easily digestible for the reader, as basal deuteration levels varies peptide-to-peptide and would not be data useful for analyzing the SIRT6:NCP interaction. All presented peptides had p values less than 0.05; thus, the HDX differences are all statistically significant. The details are in the Materials and Methods section, and a more detailed description is available in the Chalmers, et al. reference[18].

9) Smaller points:
line 173 – claim that mutated residues contribute to nucleosome binding; it would be good to mention that the contribution can be indirect.

Thank you for the suggestion. Indeed, this cannot be ruled out and we have expanded our analysis. The change (Lines 194-197) is reproduced here:

"The moderate change in affinity suggests that the HDX changes in the NBR loops represent either an indirect structural change, or a weak binding contribution that does not supply direct hydrogen bonding."

10) line 181 – an antibody is very, very bulky, can shield other regions and disrupt folding. The result thus doesn't really show involvement of the N-terminal helix in binding. More conclusive would maybe be a competition experiment with N-terminus as free peptide.

We respectfully disagree with the reviewer's interpretation. Only a small antibody region relative to the entire antibody contacts the epitope. The tight specificity of antibodies is commonly applied in supershifting experiments, which are well-established approaches to demonstrate complex formation. In fact, the antibody to the SIRT6 C-terminal region is just as bulky, yet supershifts the 100 nM and 300 nM SIRT6:NCP complexes (Supplementary Fig. 4c). To our point, a very specific N-terminal antibody disrupts the interaction with nucleosomes.

11) Figure 2e – cannot see any effect, only in the numbers?! And even there, at later times differences are minimal – how often was this reproduced?!

We made many mutations in this region, and the majority of these produced unstable proteins that were insoluble and thus not available to test our hypothesis. This was also indicated in the text (Lines 186-189). However, we were able to generate the SIRT6$^{(AAA)}$ mutant, which yielded stable protein with moderate effects on NCP binding (~7 fold) (Fig. 2e) and the predicted decline in deacetylation activity compared to wild-type SIRT6 (now in Supplementary Fig. 3c). The immunoblots were quantitatively assessed using fluorescent antibodies, so we are confident in the reported numbers (n=4).

12) Is the SirT6 C-terminus also unstructured in the full-length context? Are there, for example, CD data for full-length and C-terminal truncated protein?

Our NMR experiment with the CTD alone shows that the domain is inherently disordered (Fig. 4a), and we have found no reason to suspect that this would change in the full-length context. The protein does not behave well enough at the very low ionic strength and other buffer conditions needed for circular dichroism. Nevertheless, the first 25 amino acids of the CTD are visible in the SIRT6 crystal structure, where they are disordered[19]. The construct used for crystallography actually extends another 24 amino acids, but these residues are not visible, likely because they are also disordered. This is reinforced by bioinformatic predictions of full-length SIRT6 structure, which strongly suggest that the CTD is intrinsically disordered (Fig. 3a).

REVIEWER 2

1) In this manuscript, Liu et al., proposed the mechanism by which human SIRT6 interacts with the nucleosome. SIRT6 is a NAD+-dependent histone H3 lys9 deacetylase involved in longevity. Using biophysical and biochemical tools, such as EMSA, FRET, HDX, and mutational studies, authors claim that SIRT6 binds to the nucleosome via two asymmetric binding sites with 2:1 stoichiometry and showed that high affinity binding is driven by the association of the C-terminal disordered region of SIRT6 with the nucleosomal DNA. Although the authors proposed an interesting model by which Sirt6 recognizes the nucleosome, experimental evidence they provided was either indirect or insufficient to support their proposed mechanism without ambiguity. Therefore, I believe the authors must present more direct and solid evidence to support their model to publish their work.

Thank you for recognizing the uniqueness of our model and for these suggested improvements.

Major issues

The main interaction between Sirt6 and the nucleosome seems to be driven by the C-terminal disordered region (CTD) of Sirt6, which contains several positively charged residues. The authors also mentioned that this Sirt6 CTD and the nucleosomal DNA interactions are the sequence non-specific. This is further supported by Suppl. Fig. 2a and 4b, where free DNA also interacts with Sirt6 and disappears in EMSA. Altogether, evidence indicates that Sirt6 CTD interacts strongly (several nM affinity level) to the nucleosomal DNA in a non-specific

manner. This raises the concern that any interaction experiment performed here was mainly driven by CTD-DNA interactions, which might be independent to Sirt6 and nucleosomes.

Our data shows that the interaction between SIRT6 and NCPs is multivalent, and not dominated by the CTD-dependent interaction. In fact, C-terminal deletion mutants still harbor ~200 nM affinity for nucleosomes (Fig. 3c). We also examined direct binding between 601 DNA and the CTD. The K_D is ~400 nM, compared to 13 nM for full-length SIRT6 and nucleosomes (Fig. 1a, 4c). Thus, there are certainly CTD-independent interactions that drive binding. Importantly, our gels generally do not have much free DNA; hence, the CTD-driven effects apply to nucleosomal DNA binding, not free DNA.

We do not expect that in cells, a domain with a ~400 nM affinity for DNA would be a dominant mechanism for nucleosome binding; rather, the 13 nM composite affinity for nucleosomes via multivalent interactions is the most physiologically relevant model. This is consistent with our overall model of the CTD as a domain that supports binding, but not as a sole or dominant mechanism.

1b) More specifically, Sirt6 CTD can interact with the nucleosome in a random manner via the nucleosomal DNA . Authors state that "We propose that the promiscuous DNA-binding function of the CTD, which can accommodate multiple forms of DNA, allows the protein to rapidly find nucleosomes within nuclear confines." on page 16-17. But, this also means that Sirt6 can bind any part of the genomic DNA, not restricted by the nucleosome.

Thank you for bringing this to our attention. We would like to emphasize that while the isolated CTD has promiscuous DNA-binding characteristics, this behavior was not observed when the domain is tethered on the native SIRT6 core, which is itself restricted in orientation when bound to nucleosomes. In addition, the domain by itself has about a ~400 nM K_D for DNA, which is likely not strong enough to easily outcompete other DNA-binding proteins on chromatin (Fig. 4c). The SIRT6:NCP interaction is much stronger (13 nM), which supports the NCP as the physiologically relevant binding partner. We have accordingly re-visited our description of the CTD regarding the SIRT6 promiscuous DNA-binding ability, and re-written the relevant part of the Discussion to be clearer (Lines 371-377):

"Interestingly, only a single SIRT6 molecule employs the CTD for binding a nucleosome core particle, suggesting that the CTD is specific for a single nucleosomal DNA region. This mechanism would be consistent with the many nucleosome-binding proteins that have preference for specific regions on nucleosomal DNA, which is inherently asymmetric and features gyres of varying curvatures[8–11]. Although the isolated CTD exhibits promiscuous DNA-binding ability, the specific binding displayed when engaging NCPs suggests that the CTD is sterically restricted as part of the full-length SIRT6 protein, preventing non-specific DNA binding."

1c) The authors also suggested that CTD may recruit other factors for its specific targeting. If Sirt6 CTD participates in nucleosome-specific recognition via other binding components, then this study should be assessed again in the presence of these factors in order to be biologically meaningful.

This paper is focused on understanding how SIRT6 interacts with the most fundamental unit of chromatin, the nucleosome. We used the Discussion to speculate about how binding to specific loci could be regulated. Figuring out the mechanistic details for how the CTD (for example) might be involved in recruiting the SNF2H and CHD4 chromatin remodellers to sites of DNA damage[20,21] is beyond the scope of this paper. Most importantly, this work provides the biochemical-biophysical evidence that SIRT6 harbors the capacity to reduce global levels of H3K9ac (Fig. 5c)[2,3], without the need to form complexes with other proteins that impact chromatin binding functions, like other HDACs.

In addition to the data in the original manuscript showing the importance of the CTD in binding recombinant nucleosomal DNA, we further demonstrate in this revised update that the module interacts with native chromatin (Lines 281-9; Fig. 5a).

"The tight binding imparted through the CTD may provide the full-length enzyme with a thermodynamic advantage to interact with chromatin substrates. Thus, to determine if the CTD-dependent binding mechanism applies to native chromatin, we incubated SIRT6 or SIRT6$^{(1-301)}$ with chromatin from lysed HCT116 cells, then subjected the cells to MNase digestion. In the presence of full-length SIRT6, a substantial fraction of chromatin was strictly digested into mono-nucleosomes compared to negative control, whereas SIRT6$^{(1-301)}$-associated chromatin did not exhibit an altered digestion pattern (Fig. 5a). Thus, the CTD on full-length SIRT6 increased the nuclease sensitivity of poly-nucleosomes, which suggests that the domain imparts SIRT6 with direct, high affinity binding with native chromatin."

a

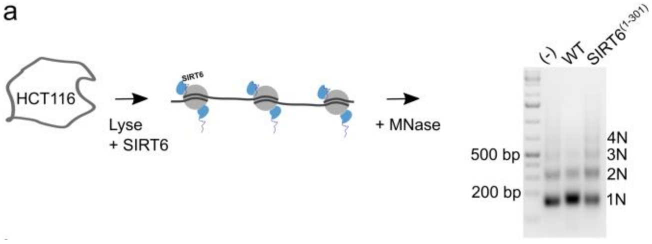

2. Authors performed the HDX experiment to identify the interface between Sirt6 and the nucleosome. I think this is a valuable approach; however, the HDX result does not match well with the current model that the authors proposed. For example, the authors proposed that the NBR region of Sirt6 interacts with the acidic patch region of the nucleosome. However, histones H2A and H2B at the acidic patch region did not show the protection upon Sirt6 interaction. The key supporting data is Sir6AAA (triple mutations of S45A/N206A/D208A), which shows seven fold decrease in binding (however, still fairly tight binding with Kd=97nM). I believe seven fold decrease with triple point mutations indicates a mild effect. Probably, affinity measurement using CTD-deleted Sirt6 1-292 with this triple mutation (AAA) may provide a more realistic affinity change between NBR and the nucleosome (without considering the effect of DNA-nucleosome interaction by CTD). In parallel, mutations in acidic patch residues to monitor the affinity change would be necessary . The LANA peptide competition assay is, in my opinion, indirect evidence and LANA binding can sterically restrict the binding of Sirt6 interaction broadly toward the nucleosome. I don't think this can support the direct interaction of Sirt6 to the acidic patch region of the nucleosome.

The HDX experiment was indeed a valuable approach that generated testable hypotheses. The reviewer is correct that the acidic patch is not represented in the HDX data, and therefore further supportive evidence is needed. We have now produced data of SIRT6 binding to acidic patch-mutated nucleosomes (H2A residues 61A/64A/90A/92A), which demonstrate that the acidic patch is important for binding (Fig. 2a; below). We further note in the Discussion that "although we do not observe differences in HDX in histone peptides corresponding to the acidic patch residues, the lack of coverage for H2A and the long peptide lengths seen in H2B may preclude such changes" (Lines 397-399). We have also removed the extraneous data with tetrasomes, which is not a physiologically relevant SIRT6 binding partner.

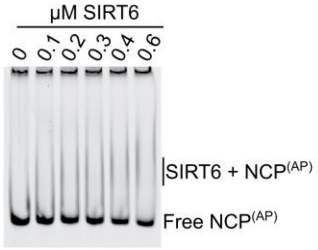

The N-terminal antibody competition experiment is consistent with the SIRT6 N-terminal helix of the NBR in binding nucleosomes (Fig. 2d). We also made efforts to generate mutants in this helix, as well as in the neighboring loops. Most mutants, however, produced insoluble proteins that were unsuitable for further follow-up experiments (Lines 186-97 below).

For the SIRT6$^{(AAA)}$ mutant that represented the loops in the NBR, we have revisited our data and agree that the binding and activity data do not support the loops binding directly to the acidic patch. Therefore, we do not suggest that residues 45, 206, and 208 may bind the acidic patch in the revised manuscript. Importantly, the data suggests that the loops do not supply direct H-bonding, and thus, any further CTD-truncated mutant experiments would not change this conclusion. We described the data in the revised manuscript as follows (Lines 186-97):

"In addition to antibody competition, we introduced strategic amino acid mutations in SIRT6. Mutations made within the N-terminal helix (34A/35A/37A) did not produce soluble enzyme, while those that included amino acids 101-107 significantly lowered the melting temperature of the protein (data not shown), making the mutants unsuitable for validation… SIRT6$^{(AAA)}$ displayed a 7-fold weaker K_D for NCPs in EMSAs ($K_{D(app)}$ = 96.6 nM) (Fig. 2e, Table 1). The moderate change in affinity suggests that the HDX changes in the NBR loops represent either an indirect structural change due to binding, or that the loops may contribute to binding NCPs, but do not supply direct hydrogen bonding."

3. Another key interacting region of Sirt6, CTD, did not show the result in the HDX experiment. Two major contacting regions, CTD and nucleosomal DNA as well as NBR and the acidic patch, are only partially demonstrated, which allows the other interpretations, such as NBR may binds other histone regions other than the acidic patch

We now provide additional data to support the importance of the CTD in nucleosome binding (Fig. 5a) and the involvement of the nucleosomal acidic patch in mediating SIRT6 binding (Fig. 2a). Our data do support that the CTD, and not the folded core, binds nucleosomal DNA (Fig. 4a,

4c). The folded core, on the other hand, is the domain that requires the acidic patch for binding (Supplementary Fig. 5d). We also clarify our statements regarding the acidic patch and the NBR to indicate that the mutants characterized suggest the importance of these residues, but that direct contact (e.g. H-bonds) does not necessarily occur.

4. Cys18 of Sirt6 is positioned in the proximity to the helix (residues 66-73) and labeling with Tide Quencher 3(TQ3), a bulky compound, would most likely break this loop and helix interaction. If this is the case, the N-terminal loop region will be flexible /mobile and the FRET data interpretation must be re-evaluated and re-considered. I'd like to hear author's opinion about this.

This is a good observation. Cysteine 18 does not appear to directly contribute much, if any, bonds with the nearest helix residues. Below, we show the distance between the cysteine with the neighboring threonine and glutamate on the helix[19].

Importantly, we noticed that the labeled protein does not affect protein stability, as assessed by differential scanning fluorometry. This is now in Supplementary Fig. 2c and shown below.

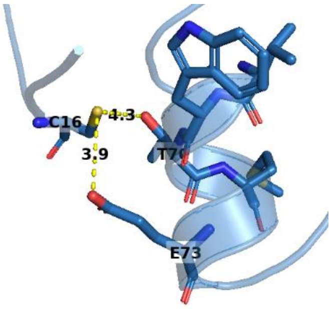

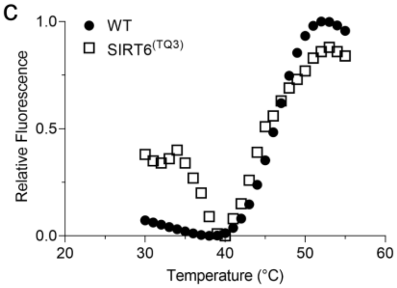

5. Although 2:1 stoichiometry of Sirt6:nucleosome seems a valid claim, how do authors envision (or distinguish) this 2:1 stoichiometry as either two asymmetric bindings on one surface of the nucleosome or Sirt6 binds on both sides of the nucleosome (as shown in cryoEM structure of Dot1L-nucleosome)? I think it is possible that one Sirt6 binds to one side of the nucleosome using CTD-nucleosomal DNA for enhancing affinity and NBR-histones for specificity (two binding sites on one nucleosome surface). Asymmetric binding was proposed based on the FRET result. But the same interpretation is possible in case with each Sirt6 binds to each side of the nucleosome (only one end of nucleosomal DNA is labeled with Cy3 and the distance of Sirt6 Cys18 in both sides of the nucleosome will display different distance). If two Sirt6 molecules bind to one side of the nucleosome, why they don't bind the other side of the nucleosome?

Thank you for bringing this up, as this is indeed an important consideration for our model that we did not address in the original manucript. The most logical interpretation is that each SIRT6 molecule binds to a separate acidic patch, located on either side of the nucleosome disc. Please see the new Figure 6 in Point 5 to Reviewer 1. We note that the LANA peptide disrupts binding to both SIRT6 molecules, so both molecules are dependent on acidic patch binding. We reason, however, that the two SIRT6 molecules do not occupy one single face of the NCP, given that SIRT6 does not dimerize under our conditions, and the acidic patch is not a large interface that

has ever been observed to bind two proteins through a single patch (Fig. 1c)[10]. We also describe our thought process in the Results (Lines 160-163) and Discussion (Lines 402-406):

Lines 160-163: "Together, these data are consistent with both SIRT6 molecules engaging the nucleosomal acidic patch. The two SIRT6 proteins likely each occupy a separate acidic patch, given that SIRT6 remains monomeric under the concentrations used, and the acidic patch is not a large interface known to bind multiple proteins simultaneously[10]."

Lines 402-406: "Because the LANA peptide disrupts binding of both SIRT6 molecules on the NCP, the simplest model is that the two SIRT6 molecules each occupy a separate acidic patch on either side of the nucleosome (Fig. 6). This mechanism is consistent with SIRT6 remaining monomeric, and with the small surface area available on the acidic patch (Fig. 1c)[10]."

6. The HDX signal change in H4 C-terminal region might be important to Sirt6 recognition and function. However, authors didn't examine any follow-up experiments. Is there any reason why?

This is a good question, as SIRT6-induced changes may have functional relevance. We note that others have observed similar, although more global, changes in the histone octamer for HP1 and SNF2H[12,13]. Their observations were further supported by intramolecular di-sulfide histone cross-linking, which restricted the function of those proteins. We decided that studying octamer plasticity was outside the focus of this manuscript, especially when we decided to focus on the role of the CTD in SIRT6 binding, activity, and cellular localization. Importantly, we explained that any perturbation at the H4 C-terminus would be due to other "SIRT6 interactions with other histone domains or nucleosomal DNA" (Line 147-148). We explore such interactions extensively in the rest of the paper. We further discuss our thoughts on the H4 C-terminus in Results Lines 142-148 and Discussion Lines 413-425 (reproduced in Point 7 to Reviewer 1).

REVIEWER 3

1) SIRT6 is an essential protein involved in numerous diverse pathways suggesting that it plays a general role in gene regulation. One way that SIRT6 exerts its function is through the deacetylation of histone tails. Histone tail post-translational modifications are an important way that eukaryotes regulate transcription of genes, and a better understanding of the biological consequences of histone modifications (and their removal) are desirable, making the subject matter of general interest to biologists. The authors present very nice in vitro characterization of SIRT6. Experiments and interpretation are generally very good.

We appreciate the positive comments about our characterization of this important enzyme.

2) However one downside is that the study falls short of adding significant biological insight into SIRT6 function. The authors probe the binding details for how SIRT6 binds a nucleosome core particle in vitro, demonstrating that two SIRT6 molecules interact with one nucleosome core particle through a two-stage process, and probe SIRT6 protein for the residues that are important for each binding stage. However, it is unclear how these observations translate to in vivo regulation.

Perhaps this is a matter of perspective. The upside of this work is that our paper explains the biochemistry behind prior biological observations, which failed to explain why SIRT6 is so tightly associated with chromatin. Prior to this report, the general consensus is that SIRT6 binds chromatin through its interaction with transcription factors[22,23]. Our results provide compelling evidence that SIRT6 is a bone fide nucleosome-binding enzyme. SIRT6 is the first described HDAC that is directly capable of specific and high-affinity interaction with nucleosomes. All other well-understood HDACs require multi-subunit complexes to bind chromatin. Please see our response to Reviewer #1 (Points 1 and 2). Also, we have characterized in detail the structure and function of the unique C-terminal domain, and in this revision, introduced new data that corroborates our evidence for the importance of the CTD (Fig. 5a).

3) The authors state in the abstract that: "Unique among histone deacetylases, SIRT6 possesses the capacity to tightly interact with nucleosomes for efficient activity. The implications for SIRT6 targeting and regulation are discussed" yet none of the observations in this manuscript provide any insight into the specificity of interaction, and it is unclear how the enzyme would discriminate appropriate substrate from random nucleosomes in the nucleus.

It is now obvious to us that we didn't make our point clear in the original manuscript: Our results that demonstrate SIRT6's intrinsic capacity to tightly bind and deacetylate H3K9ac nucleosomes provides a simple and logical explanation of numerous previous reports showing that SIRT6 is capable of decreasing global H3K9ac levels (Fig. 5c)[2,3,5,22]. This global effect has been documented in many labs, including ours, and is obvious even when comparing WT and Sirt6-/- MEFs. Thus, what we reveal in this manuscript is biologically relevant. In fact, investigating loci-specific deacetylation is off-topic for our paper. Yes, we agree that regulation and targeting are important concepts to address with SIRT6 (which we discuss), but our observations explain the lion's share of SIRT6 expression studies. We even go further and describe mechanisms, energetics, and binding sites, all of which will lend itself well to future work investigating how these mechanisms tie in to diverse pathways.

<u>REFERENCES</u>

1. Mostoslavsky, R. *et al.* Genomic instability and aging-like phenotype in the absence of mammalian SIRT6. *Cell* **124**, 315–329 (2006).
2. Tennen, R. I., Berber, E. & Chua, K. F. Functional dissection of SIRT6: Identification of domains that regulate histone deacetylase activity and chromatin localization. *Mech. Ageing Dev.* **131**, 185–192 (2010).
3. Michishita, E. *et al.* SIRT6 is a histone H3 lysine 9 deacetylase that modulates telomeric chromatin. *Nature* **452**, 492–497 (2008).
4. Onn, L. *et al.* SIRT6 is a DNA Double-Strand Break Sensor. *Elife* **9:e51636**, 1–26 (2020).
5. Kugel, S. *et al.* Identification of and Molecular Basis for SIRT6 Loss-of-Function Point Mutations in Cancer. *Cell Rep.* **13**, 479–488 (2015).
6. Chen, Y. *et al.* Asymmetric unwrapping of nucleosomal DNA propagates asymmetric opening and dissociation of the histone core. *Proc. Natl. Acad. Sci. U.S.A.* **114**, 334–339 (2016).
7. Ngo, T. T. M., Zhang, Q., Zhou, R., Yodh, J. G. & Ha, T. Asymmetric unwrapping of

nucleosomes under tension directed by DNA local flexibility. *Cell* **160**, 1135–1144 (2015).
8. Harp, J. M., Hanson, B. L., Timm, D. E. & Bunick, G. J. Asymmetries in the nucleosome core particle at 2.5 Å resolution. *Acta Crystallogr. Sect. D Biol. Crystallogr.* **56**, 1513–1534 (2000).
9. Edayathumangalam, R. S., Weyermann, P., Dervan, P. B., Gottesfeld, J. M. & Luger, K. Nucleosomes in Solution Exist as a Mixture of Twist-defect States. *J. Mol. Biol.* **345**, 103–114 (2005).
10. Zhou, K., Gaullier, G. & Luger, K. Nucleosome structure and dynamics are coming of age. *Nat. Struct. Mol. Biol.* **26**, 3–13 (2019).
11. Liu, H., Duan, X., Yu, S. & Sun, X. Analysis of nucleosome positioning determined by DNA helix curvature in the human genome. *BMC Genomics* **12**, 1–12 (2011).
12. Sinha, K. K., Gross, J. D. & Narlikar, G. J. Distortion of histone octamer core promotes nucleosome mobilization by a chromatin remodeler. *Science* **355**, 1–9 (2017).
13. Sanulli, S. *et al.* HP1 reshapes nucleosome core to promote phase separation of heterochromatin. *Nature* **575**, 390–394 (2019).
14. Luger, K. *et al.* Crystal structure of the nucleosome core particle at 2.8 Å resolution. *Nature* **389**, 251–260 (1997).
15. English, C. M. *et al.* Structural basis for the histone chaperone activity of Asf1. *Cell* **127**, 495–508 (2006).
16. Chavez, M. S. *et al.* The conformational flexibility of the C-terminus of histone H4 promotes histone octamer and nucleosome stability and yeast viability. *Epigenetics Chromatin* **5**, 1–20 (2012).
17. Simon, M. *et al.* Histone fold modifications control nucleosome unwrapping and disassembly. *Proc. Natl. Acad. Sci.* **108**, 12711–12716 (2011).
18. Chalmers, M. J. *et al.* Probing Protein Ligand Interactions by Automated Hydrogen/Deuterium Exchange Mass Spectrometry. *Anal. Chem.* **78**, 1005–1014 (2006).
19. Pan, P. W. *et al.* Structure and biochemical functions of SIRT6. *J. Biol. Chem.* **286**, 14575–14587 (2011).
20. Toiber, D. *et al.* SIRT6 recruits SNF2H to DNA break sites, preventing genomic instability through chromatin remodeling. *Mol. Cell* **51**, 454–468 (2013).
21. Hou, T. *et al.* SIRT6 coordinates with CHD4 to promote chromatin relaxation and DNA repair. *Nucleic Acids Res.* **48**, 2982–3000 (2020).
22. Zhong, L. *et al.* The Histone Deacetylase Sirt6 Regulates Glucose Homeostasis via Hif1α. *Cell* **140**, 280–293 (2010).
23. Kawahara, T. L. A. *et al.* SIRT6 Links Histone H3 Lysine 9 Deacetylation to NF-kB-Dependent Gene Expression and Organismal Life Span. *Cell* **136**, 62–74 (2009).

REVIEWERS' COMMENTS:

Reviewer #2 (Remarks to the Author):

I believe the authors addressed most of my key questions and concerns in this revised manuscript. However, I still believe the current data that supports Sirt6 recognizes the acidic patch of the nucleosome is weak and not conclusive. If authors don't want to provide further strong (or direct) evidence, I suggest to tune down the significance of this claim. (the gel shift assay using acidic patch mutations of histone H2A is not clear to me because of the smeared gel pattern and still, Sirt6 residues involved in the acidic patch are not determined here...)

Reviewer #3 (Remarks to the Author):

The authors have addressed several of my comments in a satisfactory manner. I have no other concerns.

In this response, we have made comments easy to identify by showing the reviewer's comments in bold italics, and our own responses in standard font.

REVIEWER 2

1) "I believe the authors addressed most of my key questions and concerns in this revised manuscript. However, I still believe the current data that supports Sirt6 recognizes the acidic patch of the nucleosome is weak and not conclusive. If authors don't want to provide further strong (or direct) evidence, I suggest to tune down the significance of this claim. (the gel shift assay using acidic patch mutations of histone H2A is not clear to me because of the smeared gel pattern and still, Sirt6 residues involved in the acidic patch are not determined here...)"

Thank you for your suggestion to continue strengthening the evidence of the acidic patch in SIRT6 binding and function. The original native gel of $NCP^{(AP)}$ binding to SIRT6 was not resolved well enough for quantitative analysis. Here, we repeated the experiment and were able to calculate the affinity of SIRT6 to the mutant nucleosome ($K_{D(app)}$ = 138 nM). This weaker affinity supports the proposed model, in which the acidic patch contributes to specific binding interactions. We note that distinct 1:1 and 2:1 SIRT6:nucleosome complexes are not observed in binding assays with this mutant. Instead, at higher concentrations, lower-affinity complexes appear to form at a much higher molecular mass. The figure and the main text (Lines 153-157) are reproduced below.

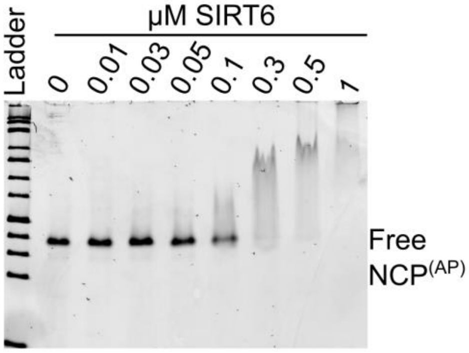

"In EMSA experiments, SIRT6 exhibited weaker binding to $NCP^{(AP)}$ than to wild-type nucleosomes ($K_{D(app)}$ = 138 nM, Fig. 2a, Supplementary Fig. 3b). Importantly, the bound species observed were slower migrating complexes, in contrast to the specific 1:1 and 2:1 SIRT6:NCP complexes previously observed (Fig. 1a)."

Furthermore, we tested the ability of our SIRT6 C-terminally truncated mutants – $SIRT6^{(1-292)}$ and $SIRT6^{(1-301)}$ – to bind $NCP^{(AP)}$. We did not detect any specific binding up to 1 μM mutant concentration, which is dramatically different from the binding experiments between the mutants and wild-type nucleosomes (Supplementary Fig. 4b). Together, the data support our conclusion that without multivalent interactions – including CTD-driven DNA binding and acidic patch binding – specific SIRT6:NCP interactions do not occur. The figure (Supplementary Fig. 5e) and main text (Lines 285-290) are reproduced below.

e

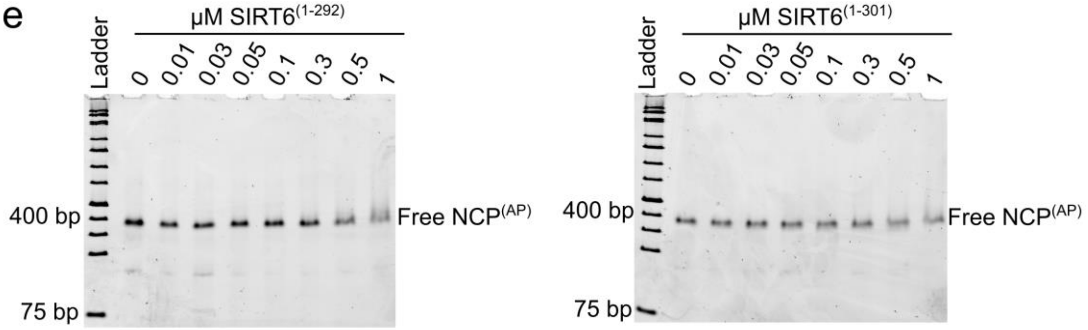

"The observation that SIRT6 can bind to both nucleosomal DNA and the acidic patch suggests that the overall complex is governed by multivalent interactions. To provide additional support, we tested if specific complexes can form when SIRT6$^{(1-292)}$ or SIRT6$^{(1-301)}$ is incubated with NCP$^{(AP)}$. Under these conditions, without available CTD-dependent DNA interactions and acidic patch docking, no specific binding was observed up to 1 μM (Supplementary Fig. 5e). Thus, multiple contact points are involved in stabilizing the SIRT6:NCP complex."

If LANA inhibits SIRT6:NCP binding, then it should also inhibit SIRT6 activity on nucleosomal substrate. Thus, we assessed SIRT6-dependent deacetylation of native chromatin substrates with and without LANA peptide. At 50 μM LANA (5x the IC_{50}; Supplementary Fig. 3c), SIRT6 is less active. The figure (Supplementary Fig. 3c) and main text (Lines 167-173) are reproduced below.

c

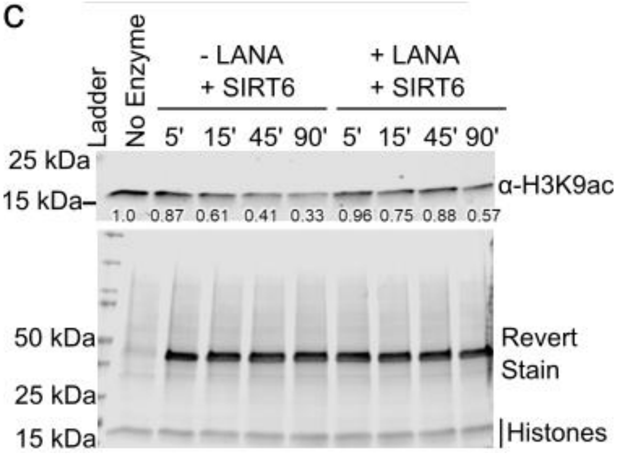

"If LANA displaces SIRT6 from nucleosomes, then one would predict that this peptide might disrupt SIRT6 activity on nucleosomes. To assess nucleosome deacetylation, we utilized native chromatin from HCT116 cells as substrate. Cells were treated with trichostatin A to preserve acetylation levels before lysis, then digested with micrococcal nuclease A (MNase A) to generate mono- and poly-nucleosomes. In the presence of LANA peptide, SIRT6 activity on HCT116 nucleosomes was decreased compared to control reactions without peptide (Supplementary Fig. 3c). Thus, binding through the acidic patch supports SIRT6 function on nucleosomes."

To ensure that LANA does not directly interfere with SIRT6 activity, we show below that LANA does not affect SIRT6 activity on a peptide substrate bearing H3K9ac, monitored in our HPLC-based peptide assay.

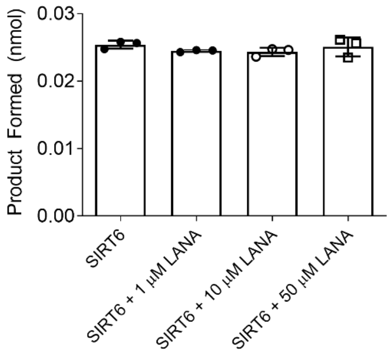

Altogether, the evidence shows that the acidic patch plays a role in binding SIRT6. We do not believe that we over-emphasize the importance of the acidic patch, as we largely state that it is a part of the overall mechanism of SIRT6-dependent function on nucleosomes.
